# Bone up on spinal osseous lesions: a case review series

**DOI:** 10.1186/s13244-020-00883-6

**Published:** 2020-06-29

**Authors:** Trinh T. Nguyen, Jarett C. Thelen, Alok A. Bhatt

**Affiliations:** 1 University of Rochester Medical Center, 601 Elmwood Ave, Box 648, Rochester, NY 14642 USA; 2 Mayo Clinic, 4500 San Pablo Road, Jacksonville, FL 32224 USA

**Keywords:** Spinal osseous tumors, Extradural spinal lesions, Vascular lesions, Neuroradiology

## Abstract

Spinal osseous neoplasms are frequently encountered and can be challenging when present as solitary lesions. Familiarity with the range of benign and malignant spinal pathology can help the radiologist formulate a comprehensive differential diagnosis. This article focuses on the spectrum of extradural spinal tumors, accounting for the majority of primary spinal tumors, by comparing the epidemiology, pathophysiology, clinical presentation, and characteristic imaging appearance of these lesions. The discussion includes the commonly encountered benign lesions, such as vertebral venous vascular malformation and enostosis, as well as malignant lesions including metastases and lymphoma. The article also includes other less-encountered primary spinal tumors such as plasmacytoma, osteoid osteoma, osteoblastoma, giant cell tumor, eosinophilic granuloma, chordoma, chondrosarcoma, osteosarcoma, Ewing’s sarcoma, and angiosarcoma. Familiarity with the characteristic imaging features can help the radiologist reach an accurate diagnosis and obviate the need for unnecessary invasive procedures such as biopsy and surgery.

## Keypoints

- A wide variety of benign and malignant entities can occur within the osseous spine.
- There can be a high degree of variability and overlap between spinal osseous tumors; knowledge of characteristic findings can narrow the differential or even synch the diagnosis.
- CT and MRI are complementary modalities, each offering different information in characterizing spinal osseous tumors.
- CT helps provide spatial details and matrix characterization.
- MRI provides contrast detail and is critical in evaluating the anatomic location and soft tissue extent of spinal tumors.

## Introduction

Most people experience back pain at some point during their lifetime, with the chief complaint of “back pain” accounting for 2.8% of all doctor visits [1]. Presenting symptoms often range from pain, weakness, to myelopathy. Many patients with these symptoms receive routine spinal imaging making this an integral part of a radiologist's daily workflow. Although the major causes of back pain are trauma or degenerative changes, spinal osseous neoplasms are frequently encountered and can be challenging when present as solitary lesions.

**Fig. 1:**
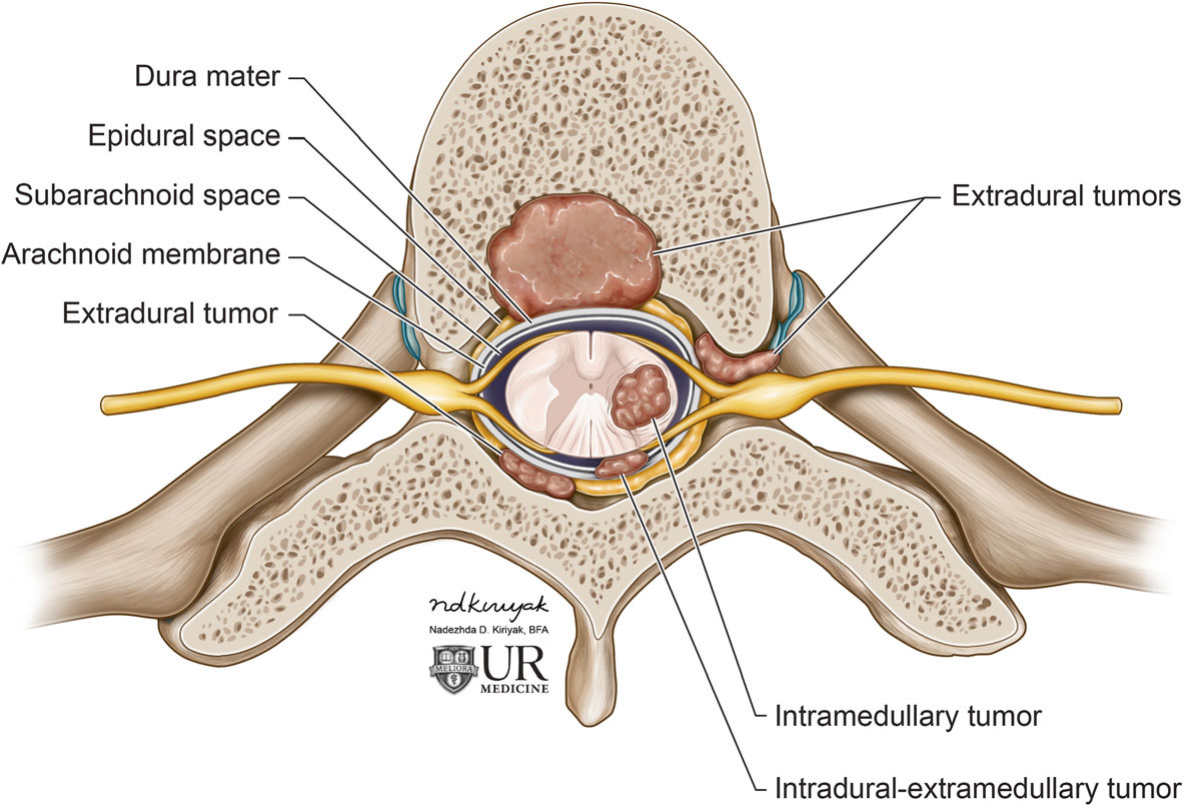
Classification of spinal tumors by location. Spinal neoplasms can be classified according to their anatomic locations, specifically extradural, extramedullary-intradural, and intramedullary tumors

Spinal neoplasms can be classified based on their anatomic locations, including extradural tumors, extramedullary-intradural tumors, and intramedullary tumors (Fig. 1). The majority of spine tumors are extradural in location, with metastases being the most common, accounting for 90% of all spinal tumors [2].

Extradural spinal lesions can involve the bones, disks, and adjacent paraspinal soft tissues. This article describes the spectrum of pathology of extradural spinal lesions, including their epidemiology, pathophysiology, clinical presentation, and imaging appearance. Lesions include metastases, lymphoma, plasmacytoma, aneurysmal bone cyst, vascular malformations, chordoma, giant cell tumor, osteoblastoma, and angiosarcoma. Spondylodiscitis is also discussed due to frequent confusion with tumors on imaging. Utilizing the diagnostic features of each lesion can help radiologists establish an accurate diagnosis and sometimes prevent unnecessary biopsy.

## Imaging modality

Although most cases of mechanical low back pain resolve with conservative treatment, radiographs are often obtained as initial screening studies. They are the most commonly ordered spinal imaging test due to their ready availability and low cost [3]. Radiographs are helpful in the evaluation of fractures, degenerative changes, disc, and vertebral body height, as well as assessment for bony density and architecture [4].

CT and MRI are advanced cross-sectional imaging modalities and more favorable for patients with more severe symptoms, neurologic deficits, or if there is suspicion of severe underlying conditions such as infection, cauda equina involvement, or cancer with spinal cord extension [4].

CT provides superior cortical bony details compared to MRI and is helpful in tumor matrix characterization. CT allows for better visualization of facet degenerative changes and fractures, particularly of the posterior elements [3]. CT is also more rapidly acquired compared to MRI, therefore more favored in the acute setting.

MRI is preferred when neurologic symptoms are present [4]. It has the advantage of not requiring radiation exposure and provides better contrast details. MRI offers superior soft tissue contrast over CT, which allows better characterization of intervertebral discs, vertebral marrow, and contents of the spinal canal [3]. MRI is critical in identifying the anatomic location and soft tissue extent of all spinal tumors.

## Benign lesions

### Vertebral venous vascular malformation (formerly known as hemangioma)

Vertebral venous vascular malformation, formerly known as hemangioma as it was thought to be a vascular tumor, is the most common benign lesion of the spine in adults [5]. They most frequently occur in the thoracic spine, followed by the lumbar spine [6]. Multiple venous vascular malformations account for 25–30% of cases [7]. These lesions are composed of vessels that infiltrate the medullary cavity around thickened bony trabeculae. Most lesions are asymptomatic and found incidentally. Occasionally these can increase in size and compress the spinal cord or nerve roots.

**Fig. 2:**
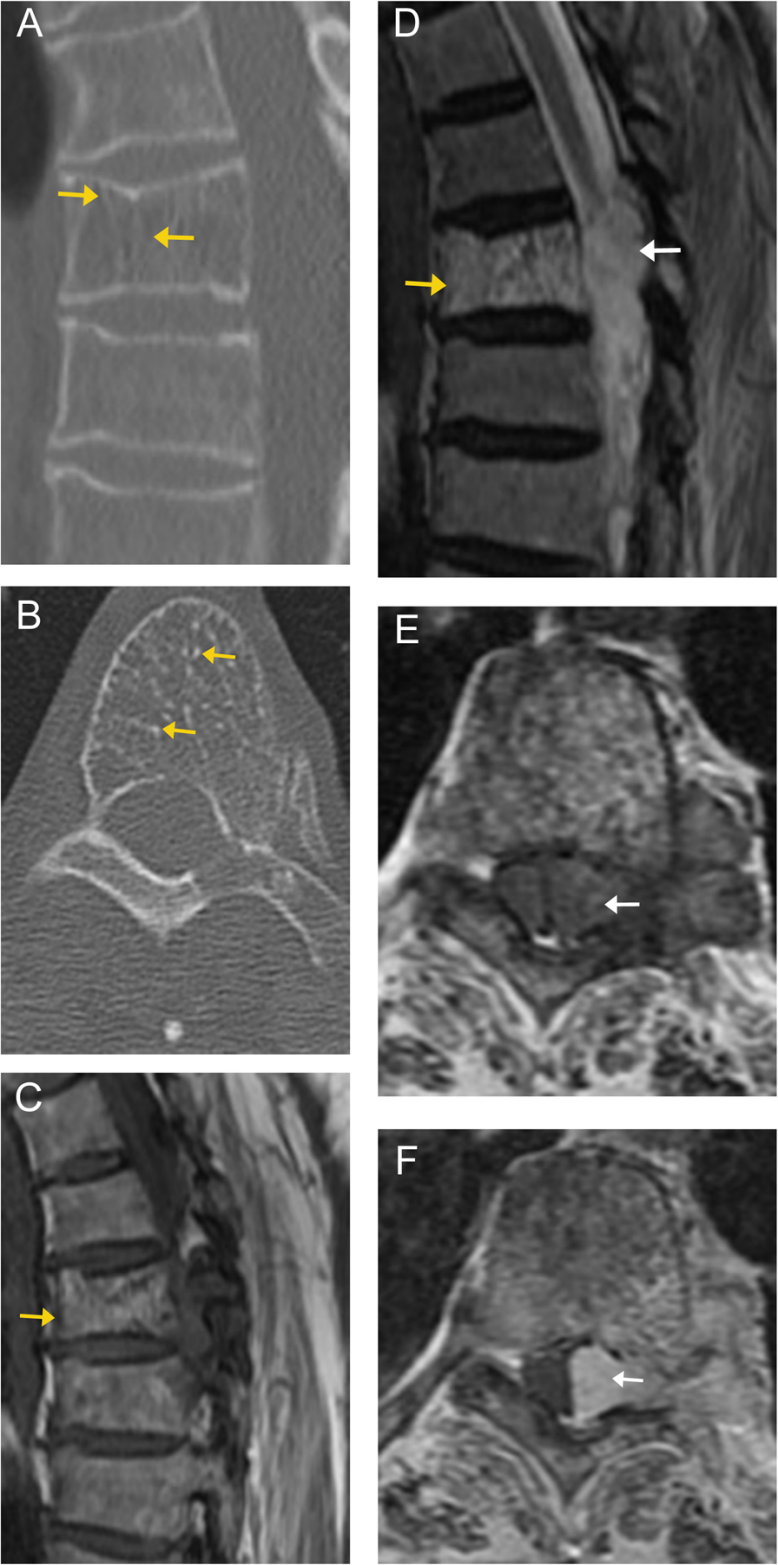
Compressive vertebral venous vascular malformation. Sagittal (**a**) and axial (**b**) CT images in the bone window of the thoracic spine demonstrate decreased attenuation of the T4 vertebral body, as well as the left pedicle and transverse process. A characteristic “polka-dot” sign is seen in the vertebral body on the axial image, with high attenuation dots representing replacement of normal cancellous bone by thick, vertical trabeculae (orange arrows) surrounded by vascular lacunes and fatty marrow. Sagittal MR images demonstrate a heterogenous, predominantly high signal lesion on both T1 (**c**) and T2 (**d**) weighted sequences involving the T4 vertebral body (orange arrows), with lobular extension into the spinal canal (white arrows). Axial T1 pre- (**e**) and post-contrast (**f**) images demonstrate avid enhancement of the lesion. Again, there is extension of the lesion from the vertebral body into the spinal canal, with resulting compression of the spinal cord

**Fig. 3:**
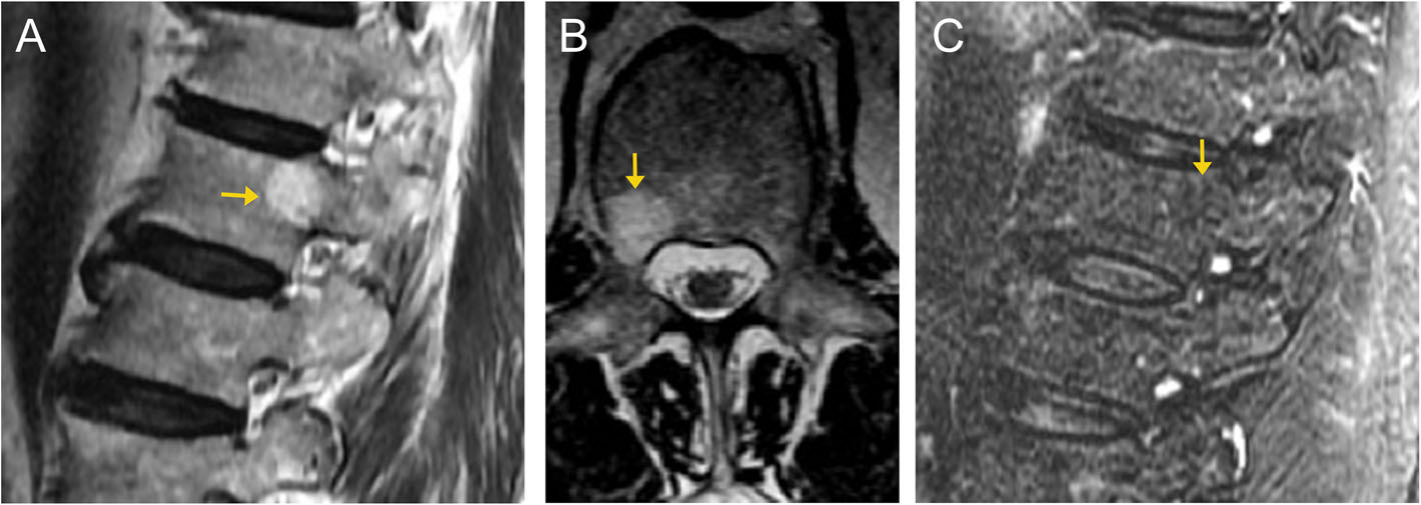
Vertebral venous vascular malformation. Sagittal (**a**) and axial (**b**) T2-weighted MR images demonstrate a well-defined lesion in the right posterolateral aspect of T12 (arrows). The lesion is hyperintense relative to the surrounding normal marrow, and demonstrates suppression (arrow) on the T2 fat-suppressed sequence (**c**). This was an incidental finding and diagnosed radiographically as a vascular malformation. Given the lack of expansion into the spinal canal or compression of the cord, unlike the prior case, this is considered a “do not touch” lesion

On CT imaging, these appear heterogeneous with hypoattenuating fat/vascular components and hyperattenuating thickened trabeculae appearing as the “polka dot” sign on axial images or vertical striations on sagittal/coronal images (Fig. 2) [8]. On MRI, these appear hyperintense compared to the adjacent marrow on T1- and T2-weighted images, with complete or at least partial dropout on fat-saturated sequences (Figs. 2 and 3). There is avid enhancement on post-contrast images [9].

Differential considerations for vertebral-confined vertebral venous vascular malformations include metastasis (typically hypointense on T1-weighted images) in the setting of known primary malignancy, especially when there is no signal dropout on fat-saturated sequences. Differential for compressive vertebral vascular malformation includes Ewing’s sarcoma, hemangioblastoma, and lymphangioma.

### Enostosis

Enostosis, or bone island, is a benign osteogenic tumor based on the WHO classification [10], is frequently encountered, and is a well-recognized entity. It represents a focus of mature compact (cortical) bone within the cancellous bone (spongiosa) [11]. It is a benign lesion, probably congenital or developmental in origin, typically incidentally found and is asymptomatic. It is often found in the pelvis, femur, and other long bones; however, it can occur anywhere in the skeleton, including the spine.

Radiographically, a bone island presents as an intraosseous sclerotic lesion with discrete margins. It is typically well-defined and homogenous and does not expand the vertebral contours [12].

**Fig. 4:**
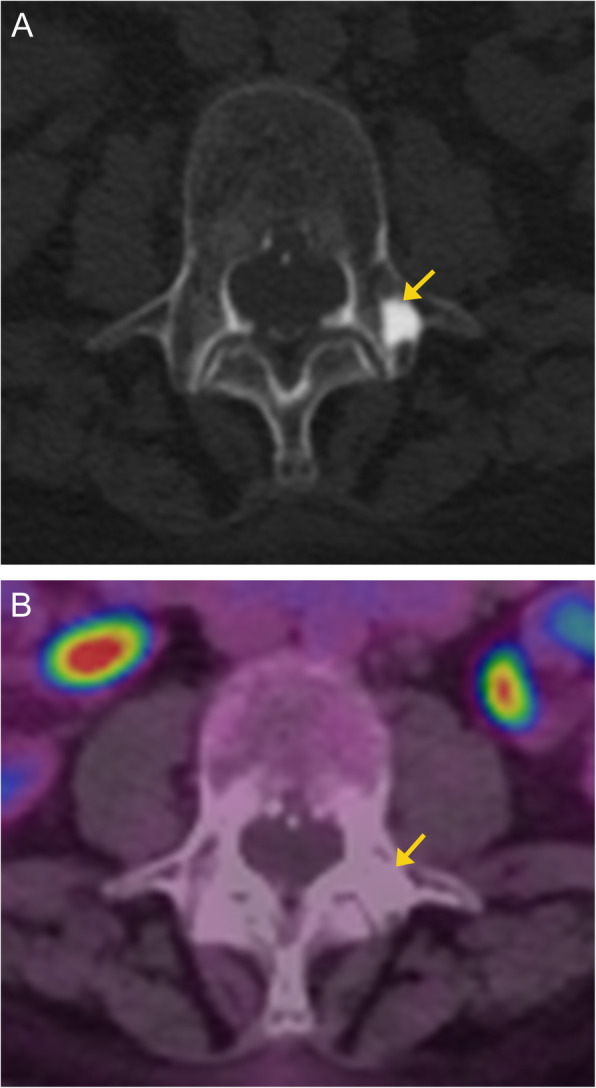
Enostosis. Axial CT image (**a**) of the thoracic spine demonstrates a focal sclerotic bone lesion with characteristic dense sclerosis and irregular and brush-like margins (arrow). No associated soft tissue component. Axial fused PET/CT image (**b**) demonstrates lack of metabolic activity of the bone lesion (arrow), confirming the diagnosis

**Fig. 5:**
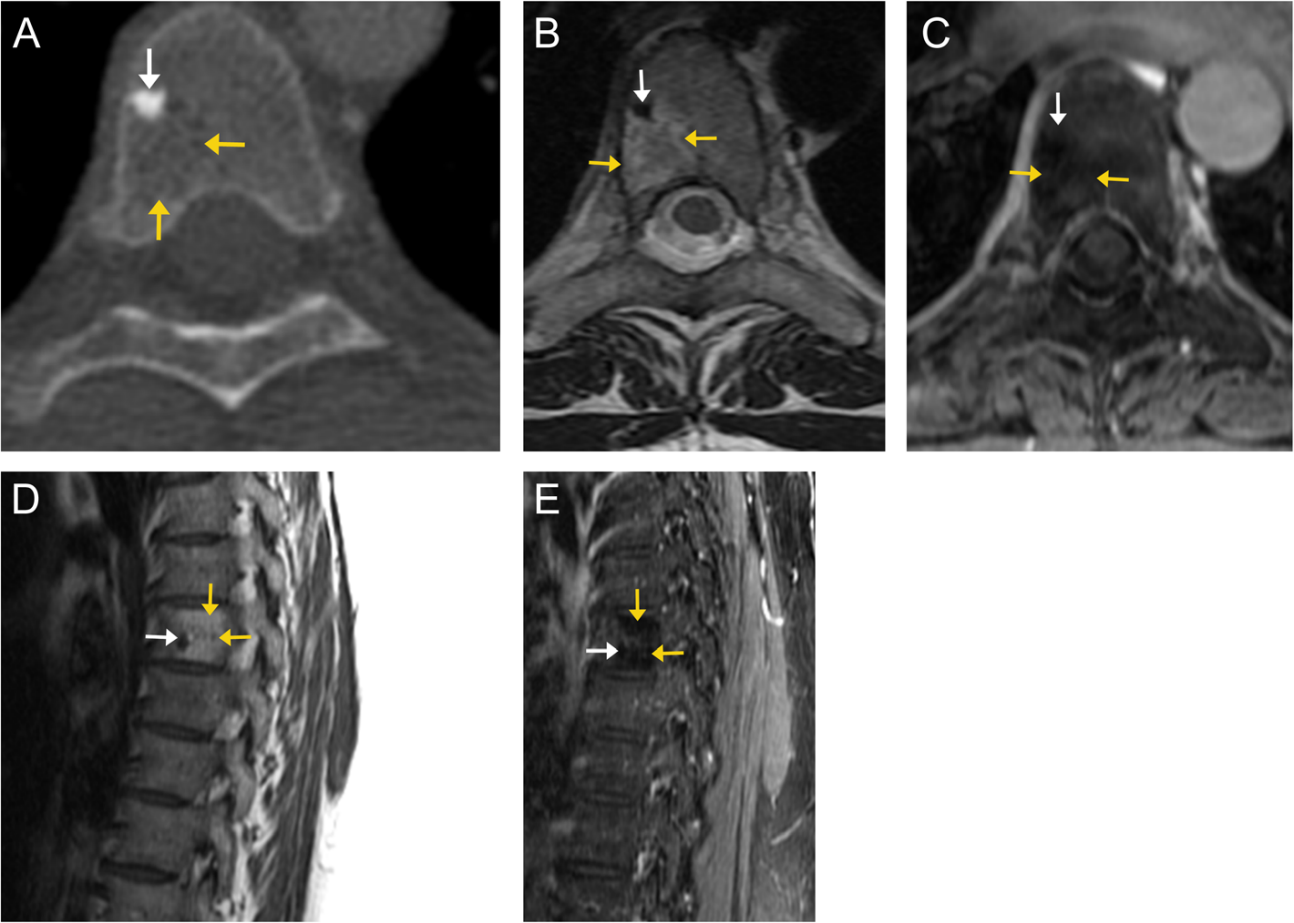
Enostosis with adjacent vertebral vascular malformation. Axial CT image of the spine (**a**) demonstrates the enostosis as a sclerotic bone lesion (white arrow). MRI shows low signal intensity like cortical bone; hypointensity on T1- (**d**, white arrow) and T2- (**b**, white arrow) weighted images, without enhancement post contrast (**c**, **e**). There is an adjacent ill-defined lesion with lucencies and intervening thickened trabeculae (**a**, orange arrows). This lesion demonstrates T1/T2 hyperintense signal (**b**, **d**, orange arrows), fat suppression (**c**, **e**, orange arrows) and no enhancement post contrast (**c**, **e**, orange arrow). This lesion is consistent with a vertebral venous vascular malformation (formerly known as a hemangioma)

On CT, a bone island appears as a high attenuation focus. Careful inspection of the borders will show radiating spicules at the peripheral margins, which blend into the surrounding bone. A distinguishing feature of bone islands is that they are usually “cold” on bone scans and PET/CT (Fig. 4) [11]. Thus, nuclear medicine bone scan is the primary modality of differentiating bone islands from more aggressive lesions. On MRI, it shows low signal intensity like cortical bone (Fig. 5). It is essential to recognize this entity and not mistake it for an ominous lesion.

### Osteoid osteoma

Osteoid osteomas are the most common benign vertebral tumors in children, accounting for roughly 10–12% [5]. They are classified as benign osteogenic tumors [10]. Vertebral osteoid osteomas occur between 10 and 20 years of age, with 3:1 male to female predilection. The most common location is the lumbar spine [13]. Patients classically present with pain that is worse at night and relieved by aspirin.

The classic radiographic appearance of an osteoid osteoma is a small circumscribed area of osteolysis, corresponding with the nidus surrounded by a rim of sclerosis. On pathological evaluation, the nidus is typically small, less than 1.5 to 2 cm in diameter, and surrounded by a rim of reactive sclerotic bone.

**Fig. 6:**
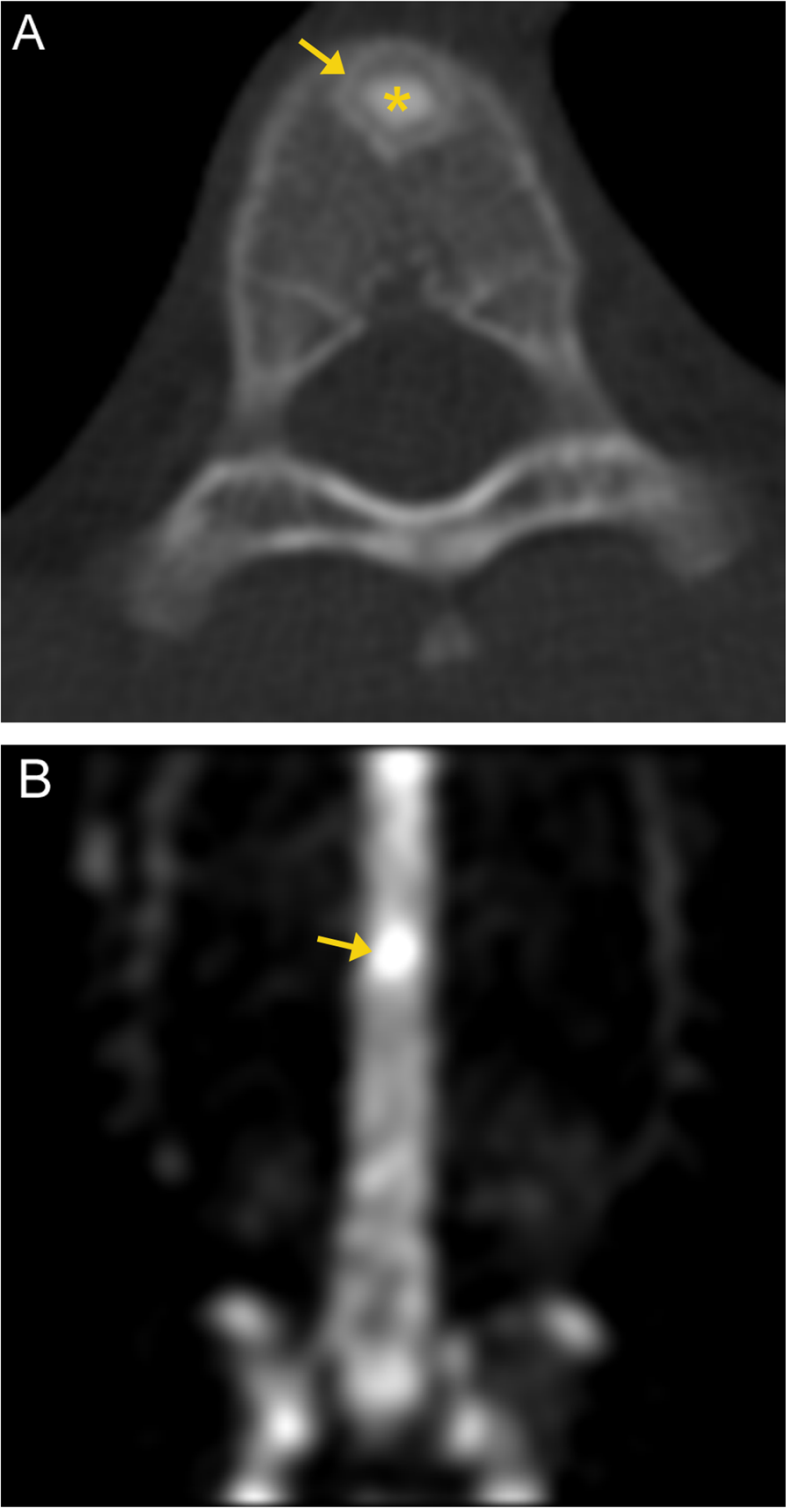
Osteoid osteoma. Axial CT image (**a**) demonstrates a 1.2-cm sclerotic nidus (asterisk) at T10. The lesion is sharply demarcated with surrounding reactive sclerosis (arrow). Nuclear medicine bone scan (**b**) demonstrates focal intense uptake at T10 corresponding within the lesion (arrow)

Osteoid osteomas typically do not grow [8]. CT is the most sensitive modality for detecting these tumors. The small nidus may or may not contain central calcification. The surrounded reactive sclerosis is better delineated on CT (Fig. 6) [9]. On MRI, the nidus usually demonstrates low to intermediate signal on T1-weighted images and intermediate to high signal on T2-weighted images [9]. Most cases will have edema in the surrounding bone marrow and soft tissue, best seen on T2-weighted images. Enhancement of the surrounding soft tissue is often seen, presumably due to inflammation [9].

The differential diagnosis for osteoid osteoma includes osteoblastoma and osteomyelitis.

### Osteoblastoma

Osteoblastoma and osteoid osteoma are both benign osteogenic tumors that produce osteoid and woven bone. About 30–50% of all osteoblastoma occur along the spine. Osteoblastoma is most commonly seen in children, in the second and third decades, with a 2:1 male to female predominance [14]. Patients typically present with dull, localized pain, as opposed to intense night pain seen with osteoid osteomas. Unlike osteoid osteoma, osteoblastoma often presents with neurologic symptoms [8]. Histologically, osteoblastoma is similar to osteoid osteoma but less organized. It is typically larger than osteoid osteoma (> 1.5 to 2 cm in diameter).

On CT imaging, osteoblastoma has three typical appearances. The most common appearance is that of an expansile lesion with multiple calcifications and a peripheral sclerotic rim. The second pattern is similar to an osteoid osteoma, with a lucent nidus surrounded by a rim of sclerosis; however, the key differentiating factor between these two is size, with osteoblastoma typically larger than 2 cm. Osteoblastoma typically grows slowly; however, the third subgroup of osteoblastoma can have aggressive growth, making it difficult to distinguish from osteosarcoma [15]. These appear as aggressive lesions, with destructive changes, soft tissue infiltration, mimicking chondrosarcoma, or osteosarcoma on imaging [8, 9, 15].

**Fig. 7:**
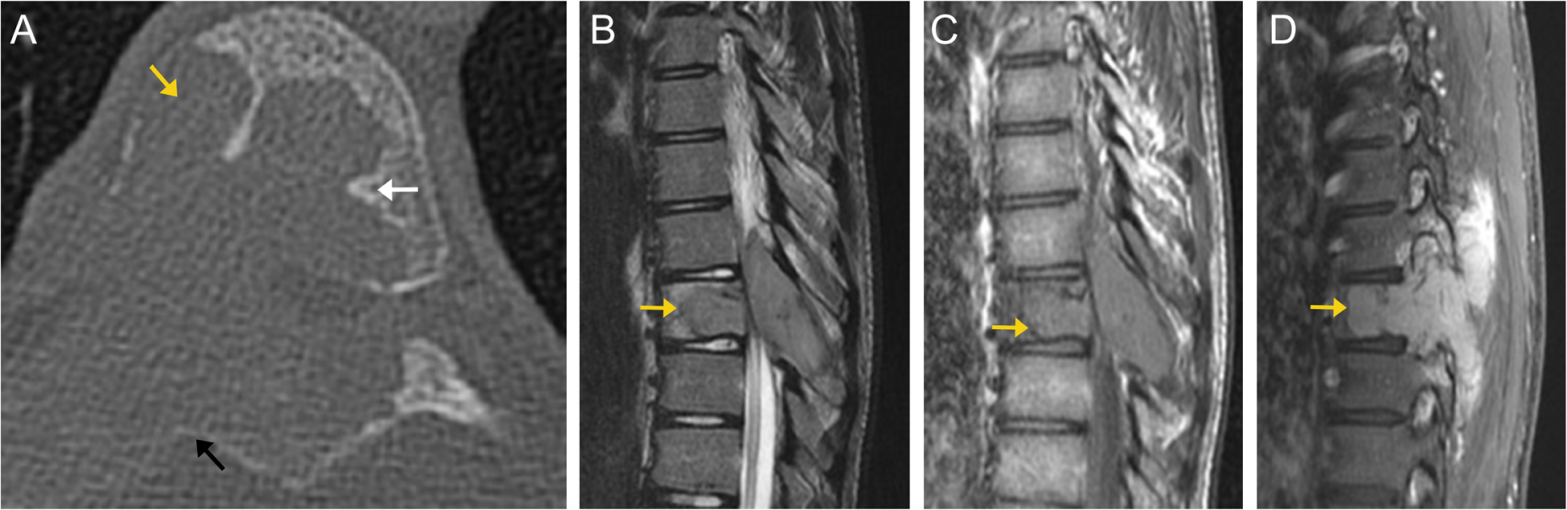
Osteoblastoma. Axial (**a**) CT image of the thoracic spine demonstrates a lytic lesion involving the T7 vertebral body (orange arrow) and posterior elements, with an expansile soft tissue component. A thin sclerotic rim can be seen along the border of the intervertebral portion of the lesion (white and black arrows). Sagittal T2 (**b**), T1 pre-contrast (**c**), and T1 post-contrast (**d**) MR images again demonstrate a lesion involving the T7 vertebral body and posterior elements (orange arrows), as well as invasion of the adjacent paraspinal soft tissues. The lesion is predominantly low to intermediate signal compared to normal marrow, and demonstrates avid enhancement on post-contrast images

**Fig. 8:**
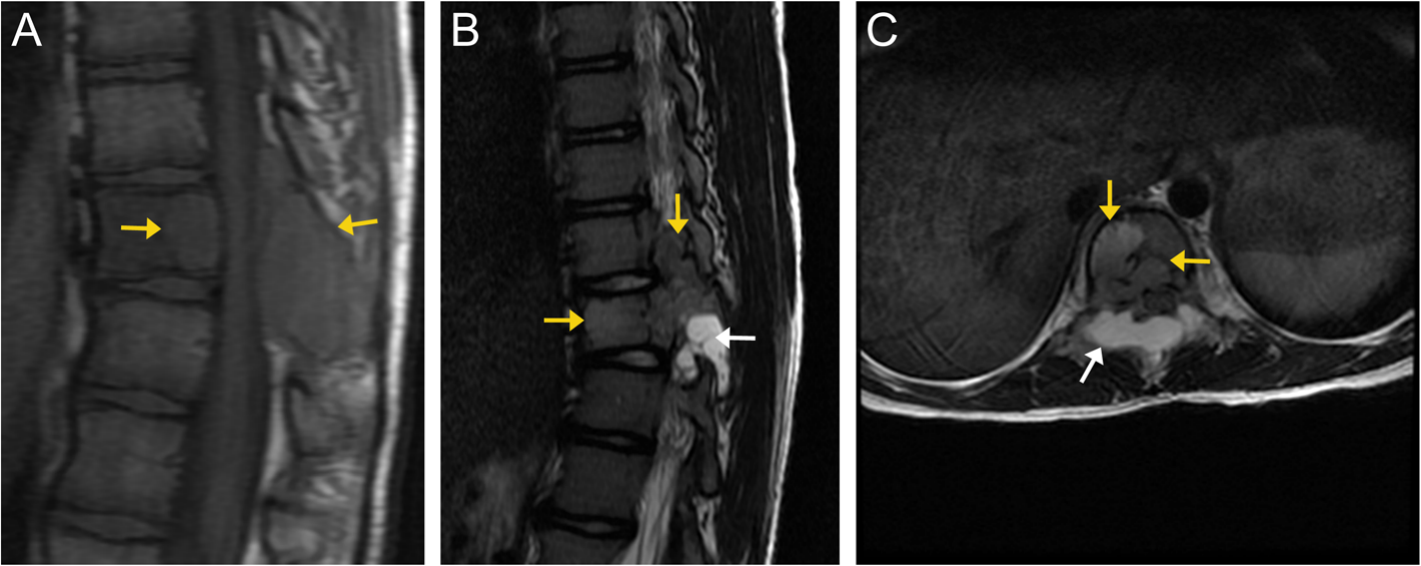
Osteoblastoma. Sagittal T1 weighted MR image (**a**) demonstrates a low signal lytic lesion involving the T10 vertebral body and posterior elements (orange arrows). The lesion is intermediate to high signal on T2 weighted images (**b**, **c**). There is an associated T2 hyperintense component, consistent with an aneurysmal bone cyst (white arrow), which can be seen in 10-20% cases of osteoblastomas

On MRI, osteoblastoma can appear similar to osteoid osteoma. They typically demonstrate low to intermediate signal on T1-weighted images and intermediate to high signal on T2-weighted images, as seen in osteoid osteomas. There may be peritumoral edema and inflammation (Fig. 7) [7]. Up to 10–20% of cases can have an associated secondary aneurysmal bone cyst (Fig. 8) [16]. These tumors can homogenously or heterogeneously enhance. Depending on the extent of involvement, they may cause neural structure impingement. The presence of a soft tissue mass suggests an osteoblastoma over osteoid osteoma [8, 9].

The differential diagnosis for osteoblastoma includes osteoid osteoma, osteosarcoma, aneurysmal bone cyst, and osteomyelitis.

### Aneurysmal bone cyst

**Fig. 9:**
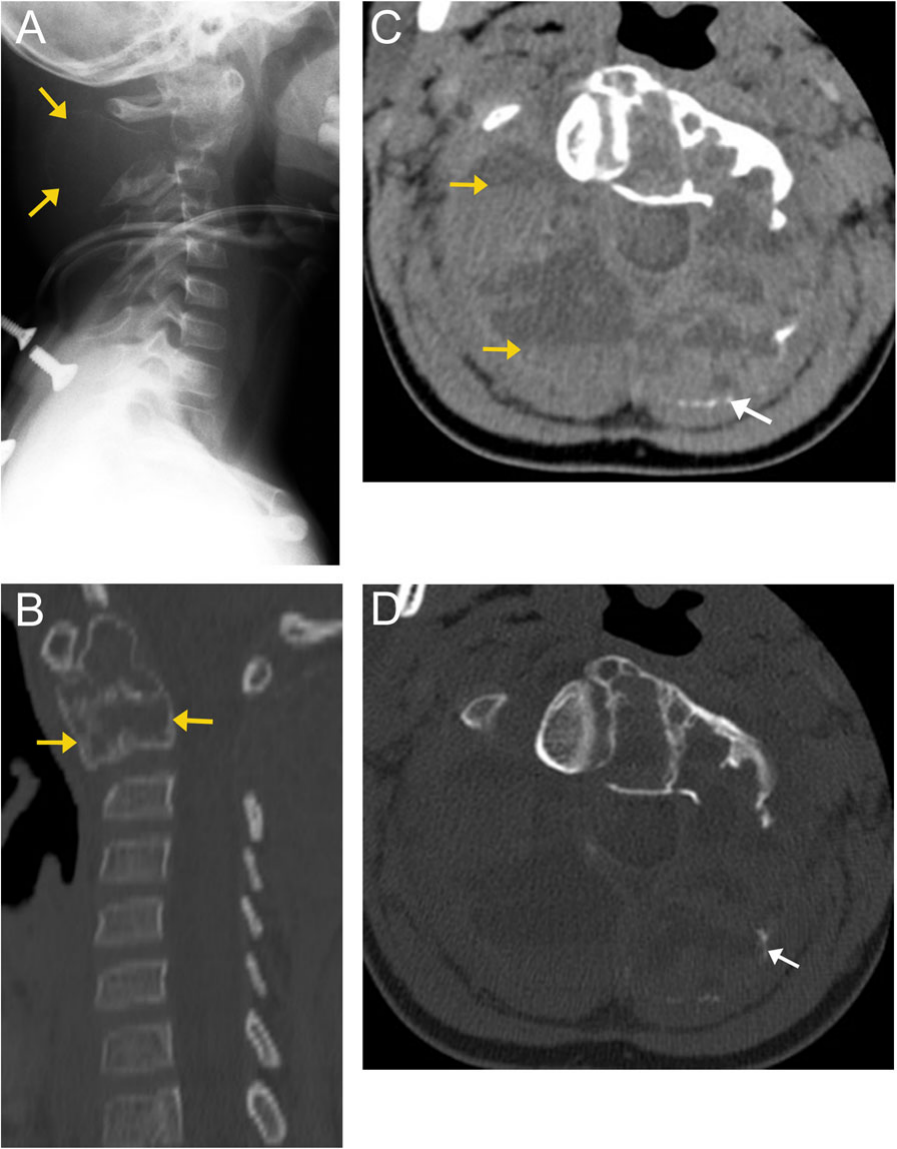
Aneurysmal bone cyst. Lateral radiograph (**a**) of the cervical spine demonstrates a large, osteolytic, expansile mass arising from the C2 vertebral body, resulting in significant bony remodeling. The mass is sharply defined and demonstrates thin sclerotic margins, also known as an “eggshell cortex” (orange arrows). Sagittal CT image of the cervical spine (**b**) confirms the lesion within the C2 vertebral body. Axial CT image in the soft tissue window (**c**) shows fluid-fluid levels (orange arrows), compatible with hemorrhage. Axial CT image in the bone window (**d**) shows near complete obliteration of the posterior arch with some areas of remnant thin sclerotic rim, "eggshell cortex" (white arrow)

**Fig. 10:**
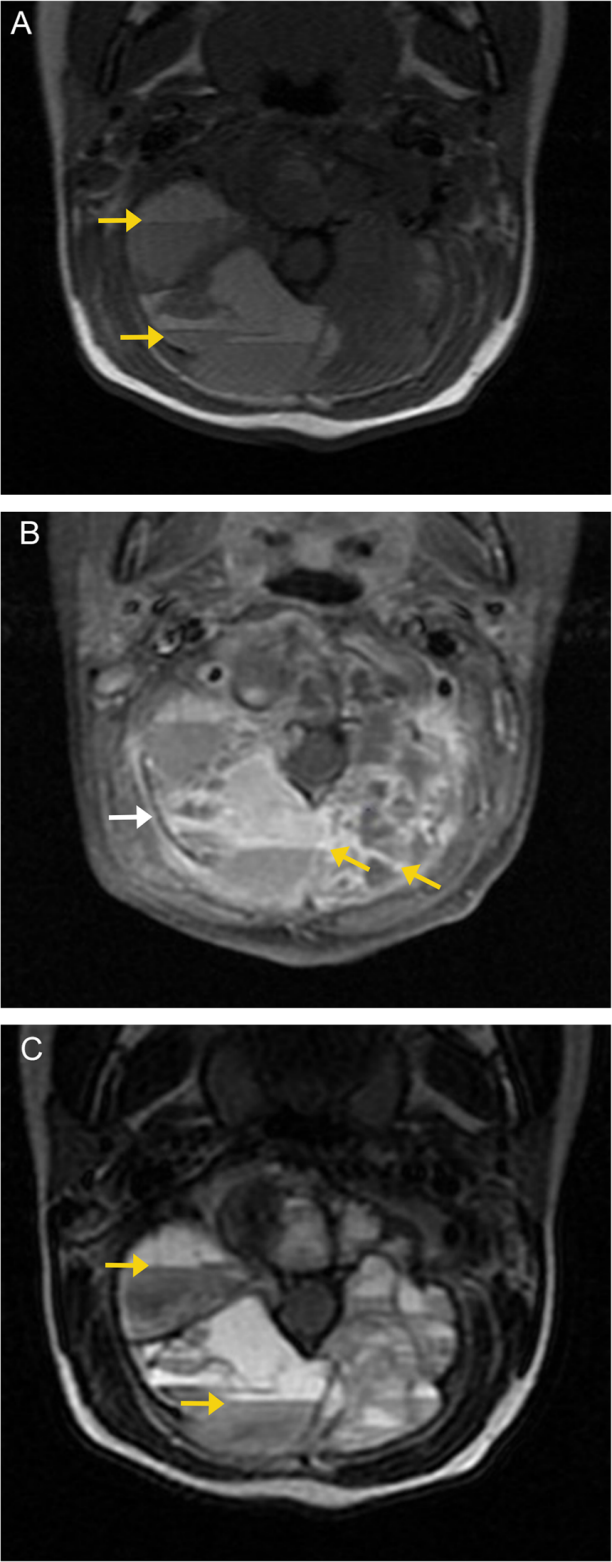
Aneurysmal bone cyst. MRI imaging in the same patient as Fig 9. Pre-contrast axial T1-weighted image (**a**) confirms fluid-fluid levels within the lesion (orange arrows), indicating hemorrhage with sedimentation of varying stages of blood products. Post-contrast axial T1-weighted image (**b**) demonstrates enhancement of the septa within the lesion (orange arrows). Note a partially hypointense rim, representing cortex (white arrow). Axial T2-weighted image (**c**) re-demonstrates a destructive lesion with fluid-fluid levels (orange arrows); varying signal intensity corresponds to differing stages of blood product degradation

Aneurysmal bone cyst (ABC), a neoplasm thought to arise from local circulatory disturbance, previously considered a benign tumor, was reclassified as a tumor of undefined neoplastic nature in 2013 according to the WHO classification of tumors of soft tissue and bone [10]. They can have a period of rapid growth, making them easily mistaken for more aggressive lesions. They are thought to arise from trauma or underlying neoplasm. These typically occur in patients less than 20 years of age [17]. Pain or neurological deficits are the most common presenting symptoms. The clinical course is highly variable. ABC can recur after curettage treatments [18]. When in the spine, these generally arise in the posterior elements, particularly the lamina, and can extend anteriorly into the vertebral body [19]. Radiographs typically demonstrate a lytic expanded bone remodeling lesion, which can involve the surrounding soft tissues. There is cortical thinning (“eggshell cortex”); CT can better demonstrate the thin cortical rim. Fluid-fluid levels are often present consistent with hematocrit levels (Fig. 9). On MRI, these appear as cysts of different signal intensities related to blood products of different stages. There is often surrounding bone and soft tissue edema, as well as peripheral and septal enhancement on post-contrast images (Fig. 10) [20].

The differential diagnosis for ABC includes lesions with fluid-fluid levels such as giant cell tumor, osteoblastoma, telangiectatic osteosarcoma, chondroblastoma, solitary bone cysts, or fibroxanthoma. These will be further discussed in the following sections.

### Giant cell tumor

Spinal giant cell tumor (GCT) accounts for approximately 7% of all GCTs [5], with the majority (~ 90%) occurring in the sacrum [21]. These lesions are cytologically benign and composed of osteoclast-like giant cells within sheets of mononuclear cells and small vascular channels. They are classified as osteoclastic giant cell-rich tumors, which are locally aggressive and may rarely metastasize or undergo malignant transformation into higher-grade sarcoma [10, 22]. The peak incidence is between 20 and 40 years of age, with a 2:1 female to male prevalence [21]. Patients typically present with pain or neurologic deficits. GCT has a poorer prognosis in the spine than the long bones, as they tend to be larger and are more difficult to excise [23].

On CT imaging, these often present as lytic lesions that cause expansion or pathologic collapse of the vertebral body [24]. Sacral GCT is usually large and destroys sacral foraminal lines, with soft tissue attenuation and a well-defined margin with a thin sclerotic rim. GCT shows no evidence of a mineralized matrix. Extension into the posterior elements and crossing of intervertebral discs may be present [9, 24].

**Fig. 11:**
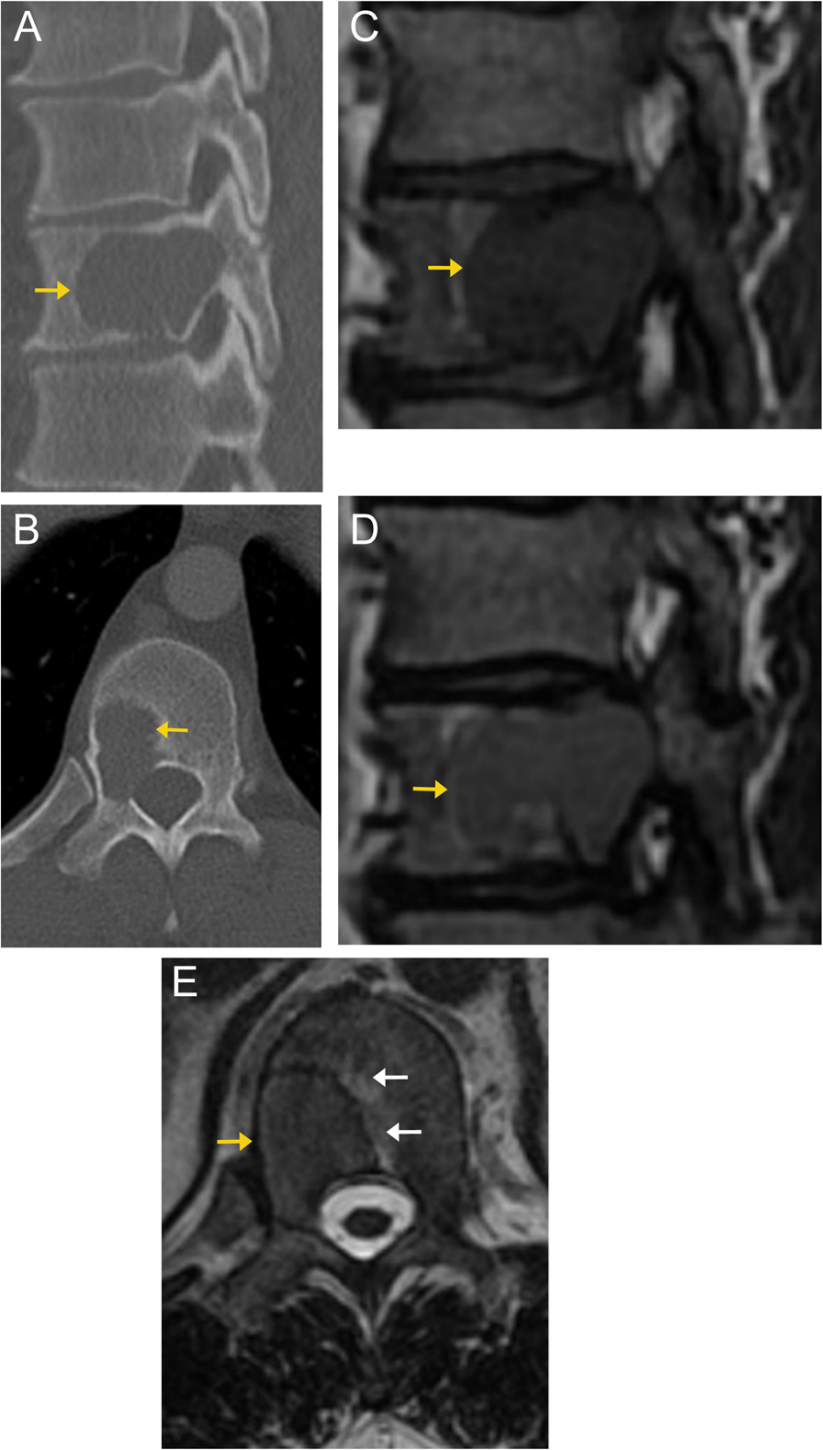
Giant cell tumor. Sagittal (**a**) and axial (**b**) CT images demonstrate a well-defined, expansile lytic lesion involving the right posterolateral aspect of the T10 vertebral body and right pedicle. No discrete sclerotic rim is seen around the lesion margins.  Sagittal and axial MR images demonstrate a homogenous, well defined mass (orange arrows) in the posterior aspect of T10, with low signal on T1- (**c**, **d**) and low to intermediate signal characteristics on T2- (**e**) weighted sequences. A trace amount of increased signal is seen in the adjacent marrow, consistent with mild marrow edema (white arrows)

On MRI imaging, they are hypointense on T1-weighted images and hypointense-to-isointense on T2-weighted images compared to the adjacent marrow signal. There may be heterogeneity on T2-weighted imaging related to fibrosis or hemosiderin deposition, which is a distinguishing feature from many other spinal tumors (Fig. 11). On post-contrast imaging, the tumor demonstrates avid enhancement of its solid components [25].

Differential considerations for GCTs include metastasis, which typically is hypointense on T1-weighted images.

### Eosinophilic granuloma

Eosinophilic granuloma (EG), a lesion of undefined neoplastic nature according to the WHO classification, is the localized form of Langerhans cell histiocytosis and most frequently occurs as a solitary lesion in bone [26]. Accounting for 12–25% of all primary spinal tumors, the lesion predominantly affects younger children of less than 15 years of age, with a male predominance. When the lesion is in the spine, it is most often in the thoracic spine, followed by the lumbar spine, then the cervical spine [26]. Patients typically present with localized pain that improves with rest, rarely with neurological complications. Other clinical symptoms may include low-grade fever, elevated ESR, eosinophilia, and leukocytosis. Treatment may include conservative management with NSAIDs or radiotherapy. Surgical decompression can be considered in patients with neurologic compromise that fail radiotherapy and have rapidly progressing symptoms [27].

**Fig. 12:**
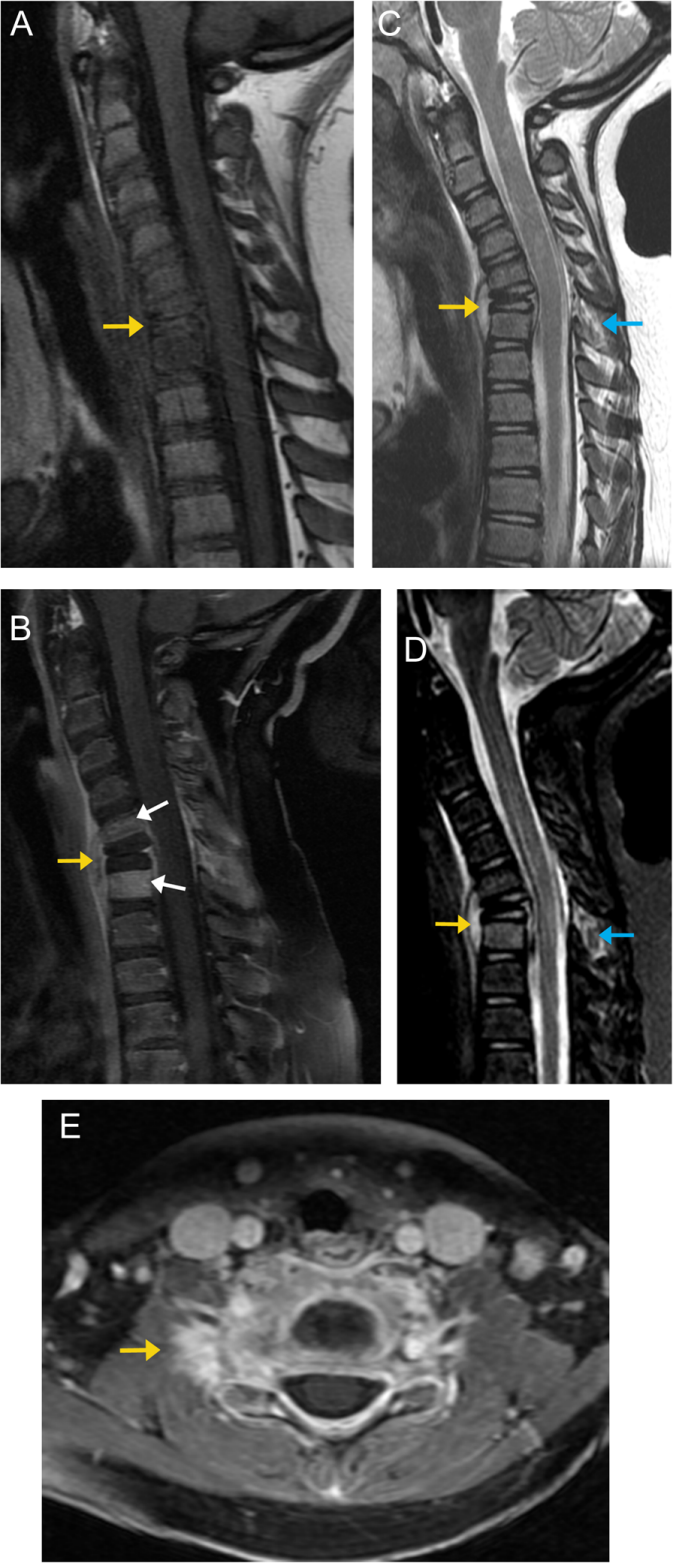
Eosinophilic granuloma. Sagittal T1 image (**a**) demonstrates suggestion of vertebral plana (orange arrow), however difficult to detect due to the lesion appearing isointense to pediatric marrow on T1. Sagittal T1 post-contrast MR image (**b**) shows an enhancing mass (orange arrows) with diffuse marrow infiltration of C5 and C7 (white arrows), and C6 pathologic vertebral collapse, sparing the adjacent vertebral endplates and discs. Sagittal T2 (**c**) and T2 fat -saturated (**d**) images demonstrate the lesion as a heterogeneously hyperintense soft tissue mass. There is edema of the posterior interspinous soft tissue, more evident on fat suppressed image (blue arrows). Axial T1 post contrast image (**e**) demonstrates asymmetric enhancement surrounding the right facets (orange arrow)

On imaging, EG destroys the vertebral body and results in the classic vertebra plana appearance. There is relative preservation of the pedicles, posterior elements, and adjacent discs. About 50% of cases demonstrate the involvement of only one vertebral level [28]. On MRI, EG appears isointense on T1-weighted images and hyperintense on T2-weighted images. Given the prominence of spinal red marrow in children, the detection of EG is relatively difficult with MRI (Fig. 12) [9].

Differential diagnosis of EG includes infectious spondylitis. Differentiating features will be discussed next.

## Pyogenic spondylodiskitis (also known as vertebral disc osteomyelitis)

Pyogenic spondylodiscitis, also known as vertebral disc osteomyelitis, is caused by bacterial infection of the spine, intervertebral discs, and extradural and intradural space. Pyogenic spondylodiskitis is not a WHO-classified lesion. However, chronic osteomyelitis confers predisposition for bone tumor and is increasingly considered a precancerous condition [10]. The imaging appearance can be confused for other osseous lesions, and therefore, it is important to include this entity in our review. The most common cause is from the hematogenous spread of infection from a remote site through an arterial route or paravertebral venous plexus. The urinary tract is the most common source. It may also occur via direct inoculation from surgery or therapeutic spinal injection, as well as contiguous spread from infected adjacent soft tissue [29]. *Staphylococcus aureus* is found in 1/3 of all affected patients [30]. Peak incidents are in the sixth and seventh decades of life. Risk factors include age older than 50 years, diabetes, chronic diseases such as renal failure and cirrhosis, acquired immunodeficiency syndrome, chronic steroid use, and intravenous drug use. Patients typically present with fever, back pain, muscle spasms, and even rapidly progressive neurologic deficits. A milder presentation, including malaise and weight loss, can also occur. Associated biochemical findings include an elevated erythrocyte sedimentation rate and C-reactive protein [31].

The primary site of infection in children is the intervertebral disc, due to its rich vascular supply. Isolated diskitis can occur in children. In adults, the intervertebral discs are nearly avascular due to involution of the intraosseous anastomoses and the rich vascular network at the vertebral disc margins. In most patients, infection is limited to the disk and adjacent vertebral bodies. However, skip lesions or multilevel involvement can occur. Infected emboli can cause infarction and infection in the metaphyses of the vertebral bodies, which can then spread to the adjacent discs. In late disease, the infection may spread into the paraspinal and epidural spaces and extend deep into the subdural or subarachnoid space [32, 33].

**Fig. 13:**
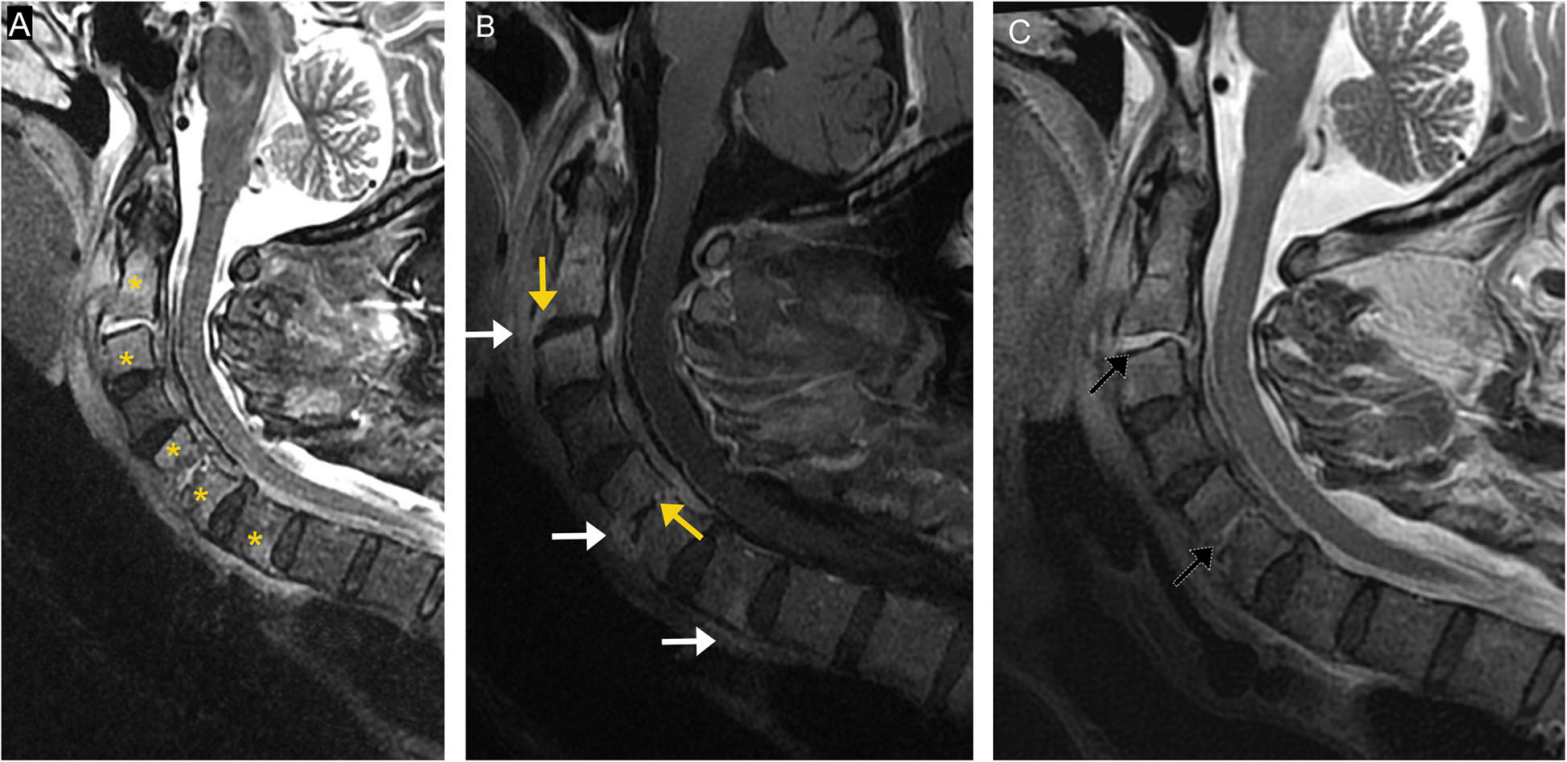
Pyogenic spondylodiskitis. Sagittal STIR image (**a**) demonstrates abnormal marrow signal of C2, C3, C5, C6, and C7 (asterisks) with diffuse paravertebral soft tissue edema. Post-contrast T1-weighted image (**b**) demonstrates corresponding marrow enhancement, disc enhancement, and endplate erosion (orange arrows). Phlegmon or abscesses are seen at the level of C2-C3, C5-C6, and C6-C7 (white arrows), as well as epidural phlegmon extending along the dorsal aspects of C2 to C7. Sagittal T2 image (**c**) demonstrates fluid signal within the disc at C2-C3 and C5-C6 (black arrows), characteristic of pyogenic spondylodiskitis

Plain radiographs are highly insensitive for spondylodiskitis, especially during its early phase. Radiographs can detect endplate irregularity and destructive changes in the late phases. CT is more sensitive to bony changes and soft tissue abnormalities. However, it is inferior to MRI, which has high sensitivity and specificity for detecting spinal infection, particularly in its early stages [32]. Sagittal MRI of the whole spine helps identify the extent of the disease. Infected areas will demonstrate increased signal intensity on T2-weighted images due to edema, with decreased signal intensity on T1-weighted images due to the replacement of marrow fat by edema. Gadolinium enhancement is more easily detectable with fat-suppressed T1-weighted images [34]. Infection typically begins in the anterior metaphyseal region of the vertebral body and spreads to the disk and adjacent vertebral body. Marrow changes typically begin at the vertebral endplates adjacent to the infected disk. Endplate erosion and vertebral bony destruction often occur. Disk involvement can manifest as increased signal on T2-weighted images, disk height loss, and disk enhancement, which is typically patchy and amorphous [32]. Associated paraspinal soft tissue involvement may be in the form of phlegmon or abscess. These typically manifest as T2 hyperintense fluid collections with restricted diffusion and peripheral enhancement (Fig. 13) [33]. MRI is not recommended for routine follow-up since imaging findings may lag or worsen despite clinical improvement [35]. Additionally, MRI may not be useful in detecting postoperative diskitis as imaging findings may be confounding and cannot reliably differentiate between pathology and postoperative changes until at least 6 months after surgery [36]. Attention to clinical history and imaging findings can accurately distinguish infection from the other spinal osseous lesions, thus expediting treatment.

There is limited utility for nuclear medicine studies despite its relatively high sensitivity due to its limited spatial resolution, low availability, long examination time, and low specificity of positive findings [37]. However, nuclear medicine scanning may be considered in patients with MRI contraindication or equivocal MRI and CT findings.

## Malignant lesions

### Vertebral metastasis

The spine is the third most common site for metastatic disease, following the lungs and liver [38]. Spinal metastases are the most common spinal tumors. Metastatic disease to the spine can involve the bone, epidural space, leptomeninges, and spinal cord.

CT can recognize bony metastatic lesions up to 6 months earlier than X-ray [39]. CT provides excellent spatial resolution and can delineate in great detail cortical destruction. However, lesions without significant osseous destruction are often missed on CT.

**Fig. 14:**
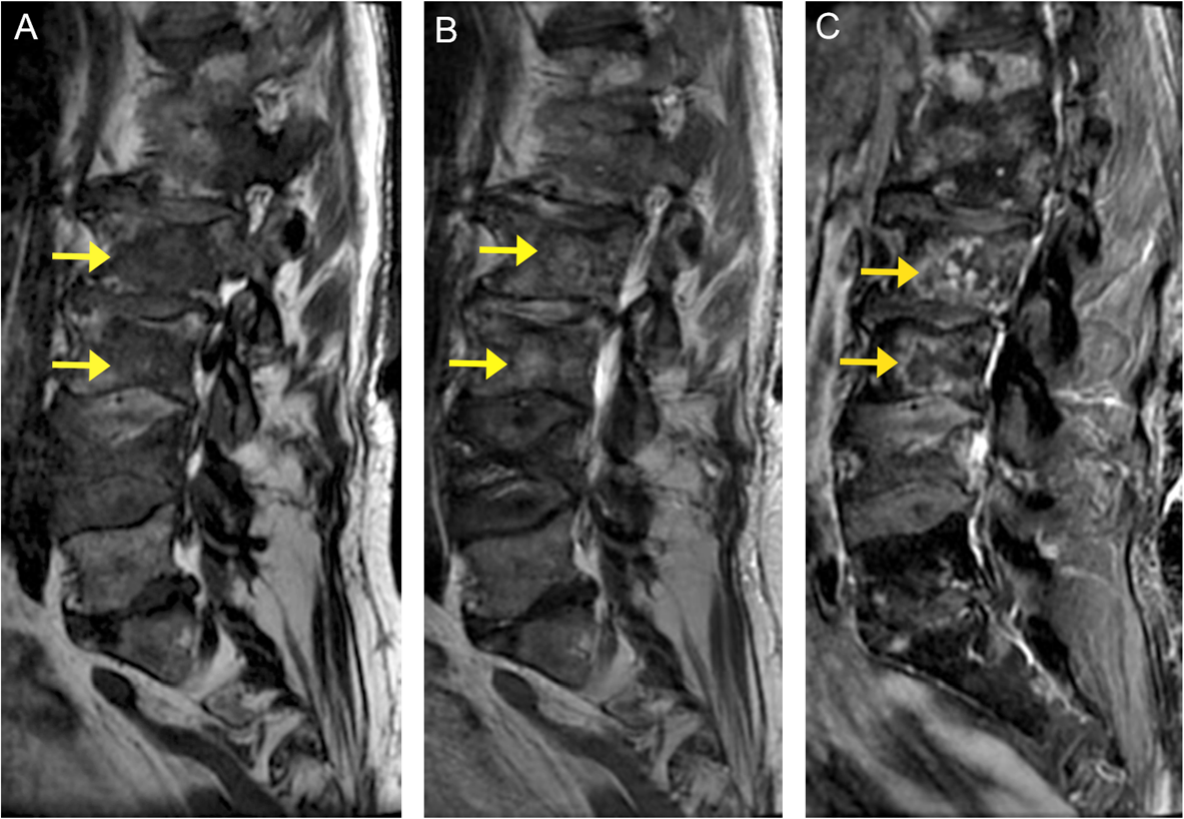
Spinal metastases. Sagittal MR images demonstrate numerous ill-defined lesions throughout the lumbar spine (arrows). These lesions demonstrate low signal on the T1-weighted sequence (**a**), intermediate to high signal on the T2 -weighted sequence (**b**), and heterogenous contrast enhancement on the post-contrast T1 fat-saturated sequence (**c**)

MRI is superior in the detection of osseous metastases compared to CT (98.5% vs. 66.2% sensitivity) [40]. MRI is the only imaging technique that allows direct visualization of the bone marrow with high spatial resolution [41]. On T1-weighted imaging, a normal fatty marrow exhibits high signal intensity. Replacement of the normal marrow by focal tumor appears as relative hypointensity to the normal marrow on T1-weighted images [42]. However, extensive or diffuse replacement of the vertebral marrow may create the impression of a normal study. On T2-weighted imaging, metastatic lesions are usually much brighter than the normal marrow due to their high water content [41]. Post-contrast images with fat suppression can increase the conspicuity of the lesion by suppressing the background bright fatty marrow signal (Fig. 14). The utility of diffusion-weighted imaging in differentiating benign from metastatic spinal lesions is controversial [41]. However, it may help evaluate response to therapy of treated tumor, with decreasing signal intensity on diffusion-weighted imaging favoring response [43].

Differential considerations for osteolytic metastases include aneurysmal bone cyst, atypical venous vascular malformations, and other primary bone tumors. Differential considerations for osteoblastic metastases include treatment-related changes, enostoses, and other primary bone tumors such as osteoblastoma.

### Plasmacytoma

Plasmacytoma is a malignant hematopoietic neoplasm according to the WHO classification [6]. It presents as a solitary lytic osseous lesion, thought to represent a focal or early manifestation of multiple myeloma. It is part of the spectrum of plasma cell dyscrasias and the proliferation of plasma cells originating from the bone marrow [44]. Solitary plasmacytoma accounts for less than 5–10% of cases and is associated with a better prognosis [45]. Plasmacytomas usually occur in the vertebra and skull [45]. Malignant plasma cells induce osteoclastic bone resorption and decrease bone formation [46].

**Fig. 15:**
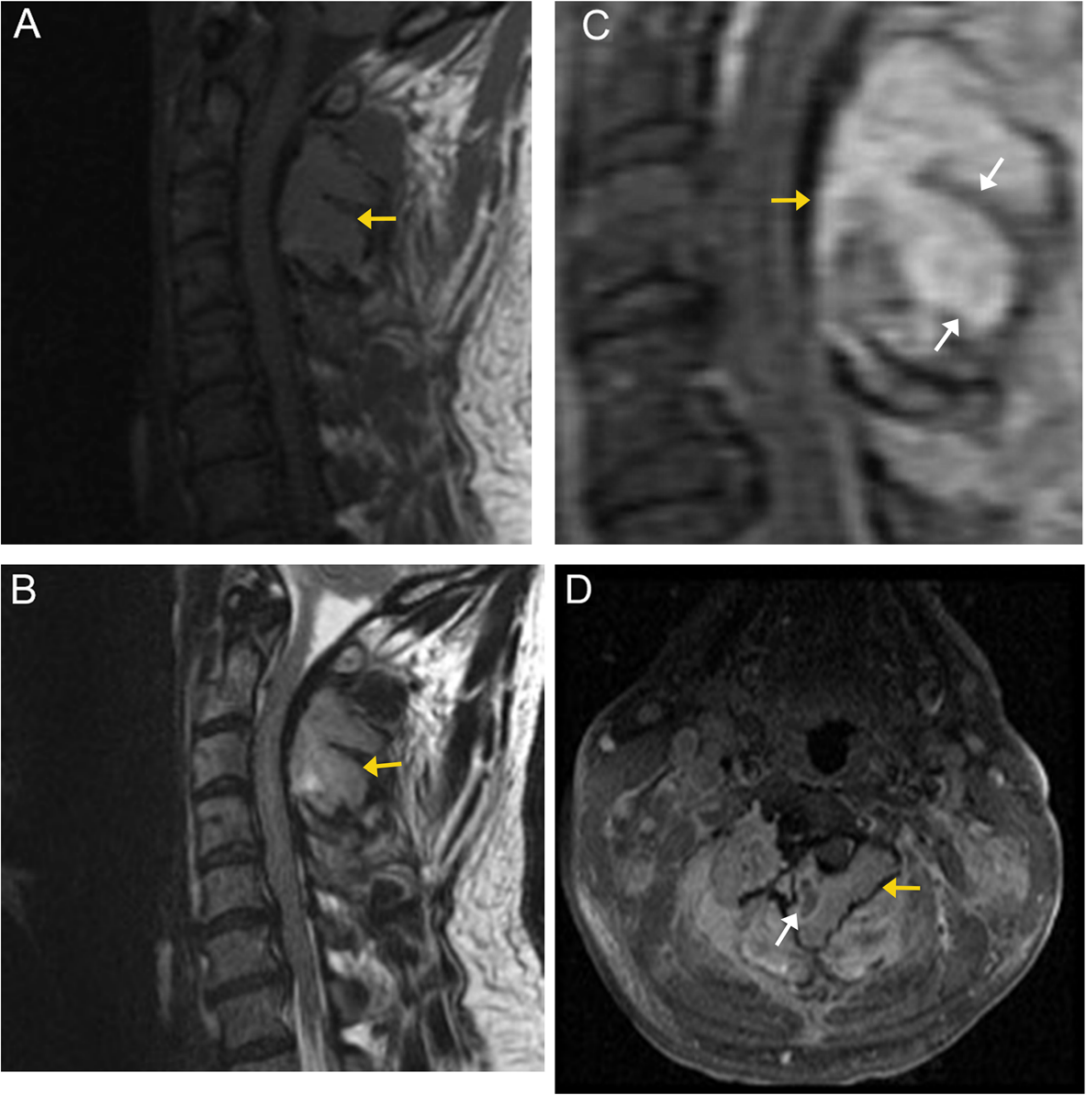
Plasmacytoma. Sagittal and axial MR images demonstrate an expansile mass (orange arrows) arising from the posterior elements of C2 and C3. The mass demonstrates hypointense signal to normal marrow on T1 (**a**), and isointense to hyperintense signal on T2 (**b**). Post-contrast sagittal T1 (**c**) and axial T1 fat-suppressed (**d**) sequences demonstrate enhancement. A few areas of curvilinear low-signal intensity are seen at the remaining thickened bony cortex (white arrows), projecting into the lesion, resulting in a “mini-brain” appearance resembling cortical sulci

CT is more sensitive than skeletal radiographs in assessing the disease extent. On CT, plasmacytoma appears as a heterogeneous mass that is predominantly lytic with partial preservation or sclerosis of the outer cortex. MRI is more sensitive than radiographs in detecting spine and pelvic disease. On T2-weighted imaging, plasmacytoma appears as a circumscribed lesion that is heterogeneously isointense to hyperintense compared to the surrounding normal bone marrow. On T1-weighted imaging, the lesion appears hypointense. There may also be linear areas of low-signal cortical struts extending into the lesion, resulting in a “mini-brain” appearance [47]. On post-contrast imaging, plasmacytomas demonstrate moderate enhancement (Fig. 15) [48]. Pathologic vertebral compression fractures can occur.

Differential considerations are metastases (which typically does not involve disc, whereas plasmacytoma may cross multiple vertebral levels), osteomyelitis, aneurysmal bone cyst, and osteoblastoma.

### Lymphoma

Lymphoma is considered a malignant hematopoietic neoplasm [6]. The spinal manifestation of lymphoma is most commonly secondary to disseminated lymphoma. Primary spinal lymphoma is relatively rare. Vertebral body and epidural lymphomas are the most common forms of spinal lymphoma, accounting for only 1–3% of all lymphoma [49]. Non-Hodgkin’s lymphoma is the most common, with the peak incidence of 40–60 years of age, and 8:1 male to female predominance [50]. Type and degree of involvement of the spine are variable with vertebral, paraspinal, and epidural patterns as most common [49]. Cord compression is present in up to 7% of cases [51].

**Fig. 16:**
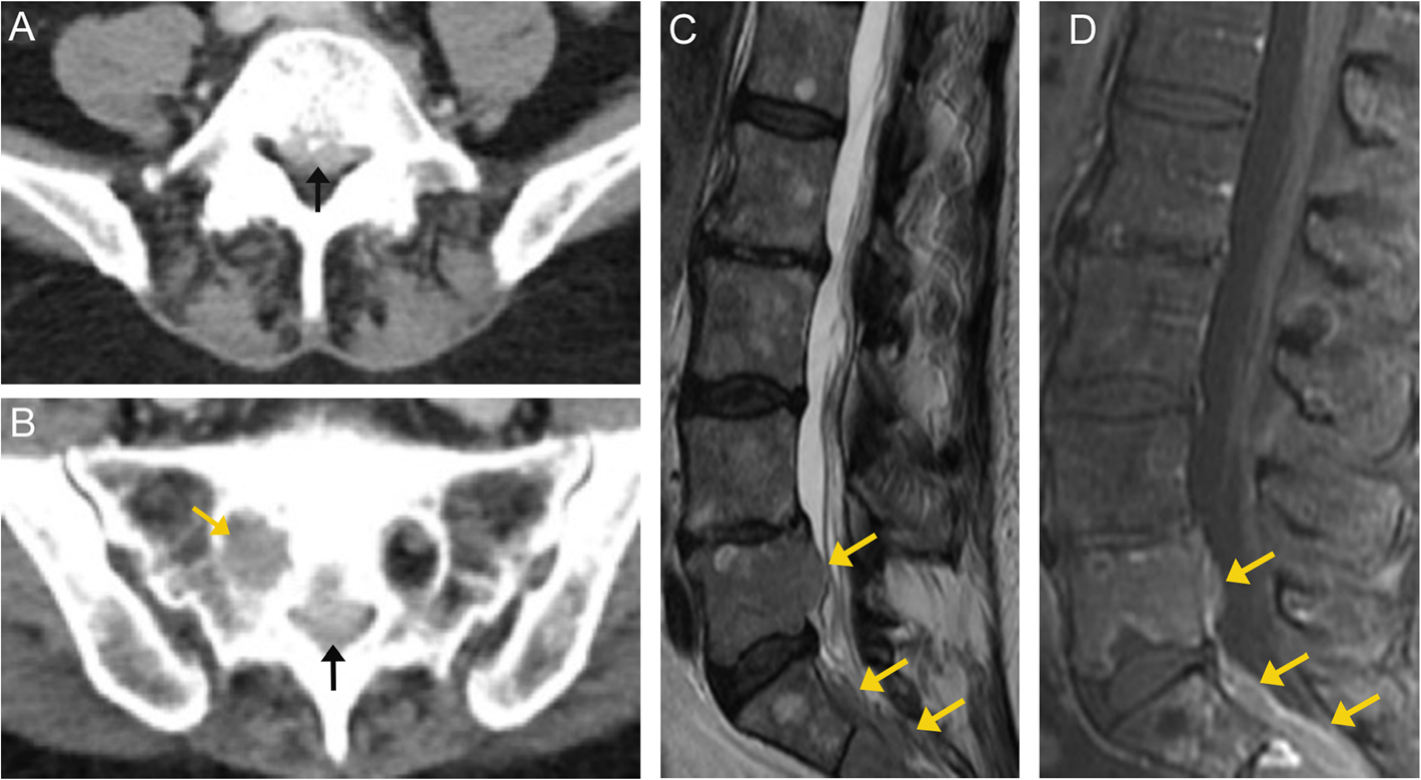
Lymphoma. Axial CT images through the lumbar spine (**a**) and sacrum (**b**) demonstrate abnormal soft tissue within the spinal canal (black arrows). Additionally, there is soft tissue replacement of the normal fat in the right sacral foramen (orange arrow). T2-weighted MR image (**c**) of the lumbosacral spine demonstrates an intermediate to low signal lesion involving the posterior aspect of L5, and another component at S1-S2 (orange arrows). There is no involvement of the intervertebral discs. Post-contrast T1-weighted fat-suppressed image (**d**) demonstrates relatively homogeneous enhancement

Imaging appearance of spinal lymphoma is relatively nonspecific, as different lymphoma subtypes may have different imaging characteristics; it is therefore important to pay close attention to history. A typical CT appearance is an epidural tumor with adjacent soft tissue or nodal mass. Osseous involvement is relatively rare [51]. MRI findings are also nonspecific. On T2-weighted images, the abnormal marrow can appear hyperintense. Contrast-enhanced T1-weighted imaging helps delineate extraosseous soft tissue involvement, paravertebral infiltration, and the degree of spinal canal and cord involvement (Fig. 16).

Differential considerations include post-treatment changes, metastatic disease, multiple myeloma, and Ewing’s sarcoma.

### Chordoma

Chordomas are slow-growing malignant notochordal tumors arising from notochord remnants [10]. They represent the second most common primary malignant tumor of the spine in adults, accounting for approximately 20% of primary spinal tumors [52]. The most common being multiple myeloma. The peak incidence is between ages 50 and 60, with a 2:1 male to female predilection [53]. Most chordomas recur post-treatment due to incomplete resection or violation of tumor capsule with seeding of the tumor to surrounding tissues. Enbloc resection is the primary treatment [54].

**Fig. 17:**
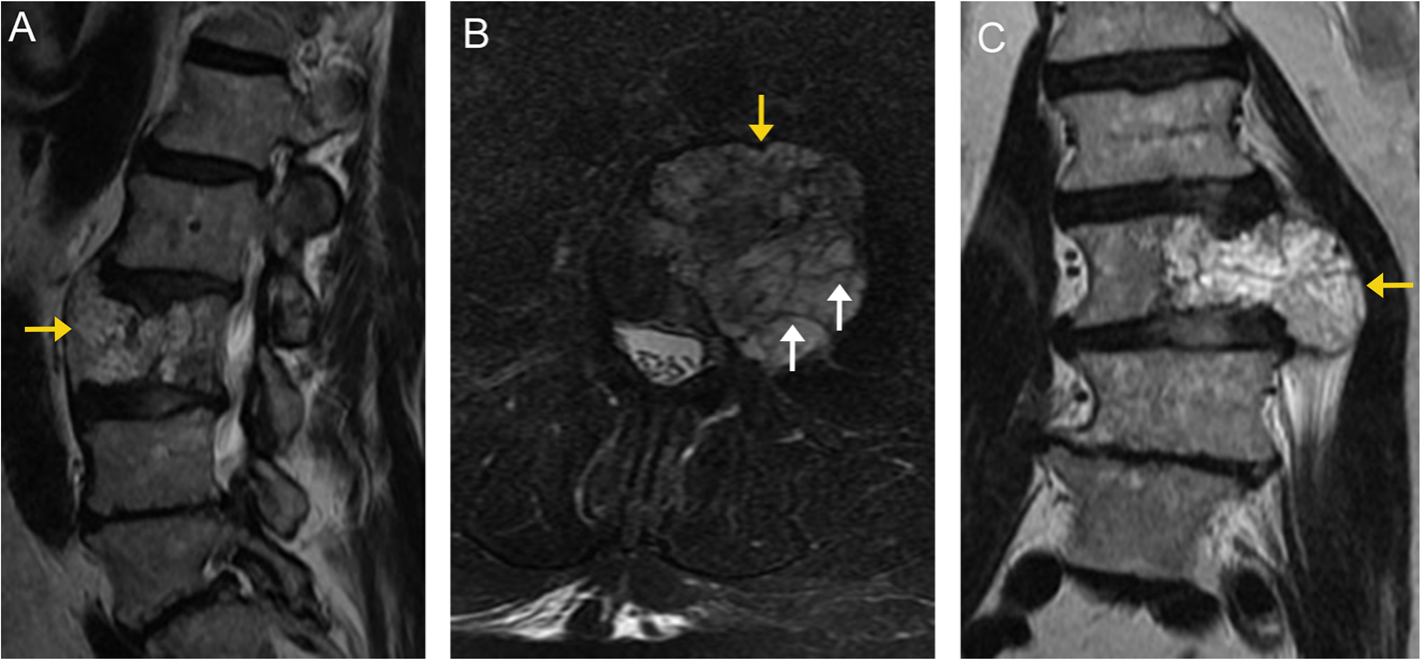
Chordoma. Sagittal T2-weighted (**a**), axial T2-weighted fat-saturated (**b**), and coronal T2-weighted (**c**) images demonstrate a heterogeneously hyperintense, expansile and destructive lesion (orange arrows) involving the L3 vertebral body. Numerous internal septa (white arrows) are seen within the lesion, creating a “honeycomb” configuration. The lesion extrudes out and over the left aspect of the veretebral body (creating a "mushroom" appearance), and displaces the adjacent paraspinal musculature

On CT imaging, they appear as midline expansile soft tissue lesions displacing the adjacent paraspinal musculature, resulting in a “mushroom” or “dumbbell” appearance. The tumor can cause destructive changes of the vertebral body and extend into the spinal canal [55]. On MRI, they have low to intermediate signal on T1-weighted images and very high signal on T2-weighted images due to their high water content. The low-signal fibrous septations on T2-weighted images form a “honeycomb” appearance. The tumor demonstrates heterogeneous enhancement on post-contrast imaging (Fig. 17) [56].

Differential considerations for chordomas include chondrosarcoma, giant cell tumor (typically more intermediate to high signal on T2-weighted images), metastases, plasmacytoma, and lymphoma. However, the midline “honeycomb” appearance is a key distinguishing feature of chordoma.

### Chondrosarcoma

Chondrosarcoma is a malignant chondrogenic tumor [10]. It is the third most common primary malignant spinal tumor, after multiple myeloma and chordoma. They account for up to 25% of all primary malignant spinal neoplasms [57]. Patients are usually between the age of 33 and 51 years, with a 4:1 male to female predilection [57]. Spinal chondrosarcoma most commonly occurs in the thoracic spine (60%). They usually arise from the posterior elements and present as a palpable mass. The most common presentation is focal pain that gradually worsens. Many patients will present with radiculopathy, myelopathy, or cauda equina syndrome [58]. Most chondrosarcomas are primary. However, secondary chondrosarcomas can arise from a preexisting benign osseous or cartilaginous tumor, such as an osteochondroma.

**Fig. 18:**
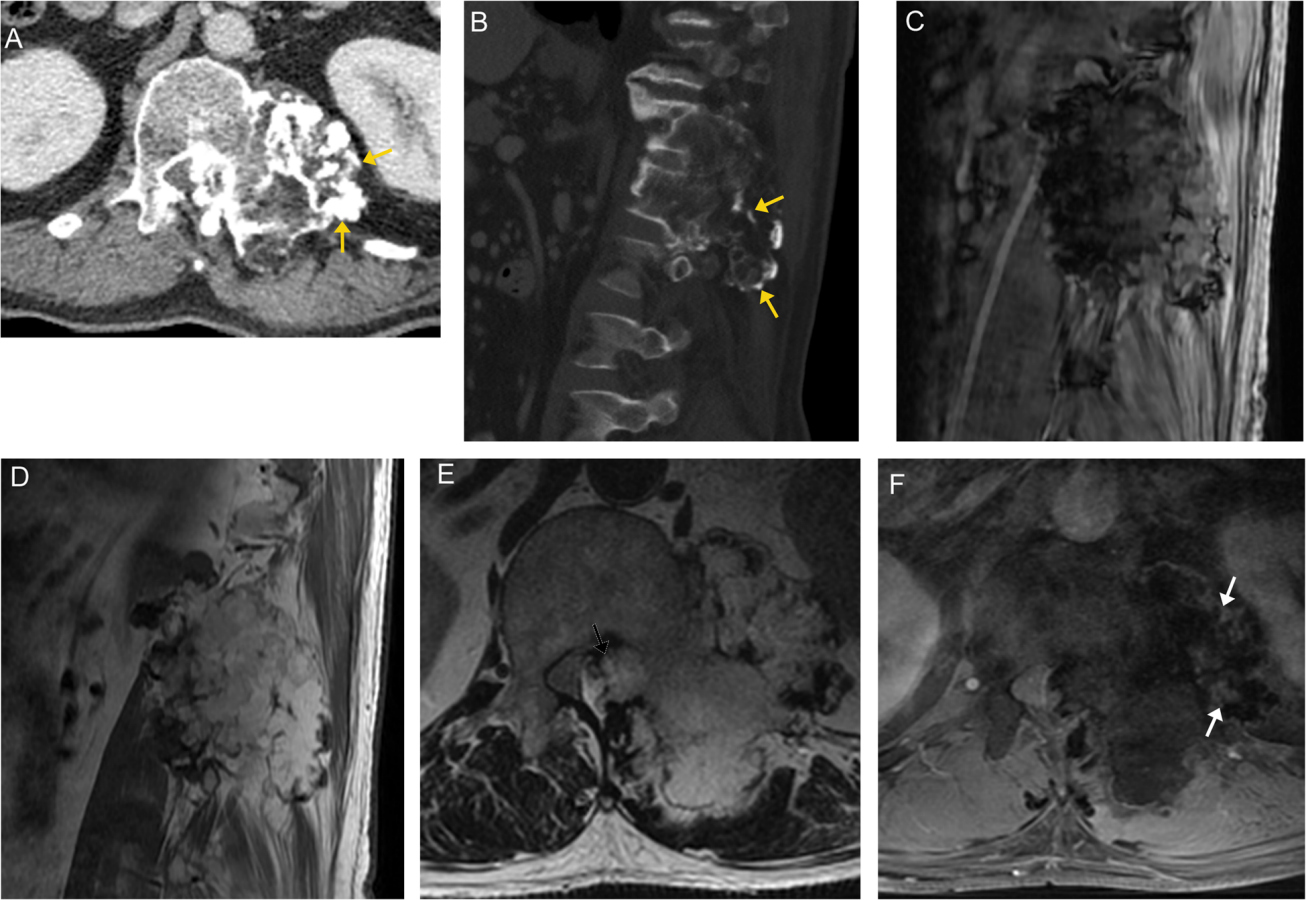
Chondrosarcoma. Axial CT image in the soft tissue window (**a**) demonstrates a mass centered at the left paravertebral region with expansile ring and arc calcifications (arrows), typical of a chondroid tumor matrix. On sagittal off-midline CT image (**b**), the tumor is seen extending over multiple vertebral levels. Sagittal GRE image (**c**) demonstrates areas of low signal within the tumor, due to its calcific components. Sagittal T1-weighted image (**d**) demonstrates variable signal intensity of the tumor. Axial T2-weighted image (**e**) demonstrates lobular, predominantly high signal intensity of the tumor due to the high-water content of hyaline cartilage. Additionally, there is epidural extension of the tumor (black arrow), displacing the spinal cord. Peripheral areas of low signal intensity are due to calcifications. The T1 fat-suppressed post-contrast image (**f**) demonstrates enhancement along the periphery and internal septa (white arrows). The non-enhancing areas represent hyaline cartilage, cystic mucoid tissue and necrosis

Radiographs of spinal chondrosarcoma typically show lytic or expansile osseous destruction. Ring and arc calcifications of chondroid matrix mineralization are present in up to 70% of patients. Extension to adjacent vertebral bodies or adjacent ribs can occur. On CT, they are typically hypodense compared to the surrounding skeletal muscles, due to the high water content of the chondroid [59]. On MRI, the chondroid component appears as hyperintense on T2-weighted images and hypointense on T1-weighted images. The mineralized cartilage appears as low signal foci [59]. Low signal septae are commonly present. The lesion demonstrates heterogenous post-contrast enhancement. Extradural extension with cord compression can occur (Fig. 18).

The chondroid calcification can sometimes be amorphous, making it challenging to distinguish chondrosarcoma from other osteoblastic bone tumors, such as osteosarcoma or Ewing’s sarcoma.

### Osteosarcoma

Osteosarcomas are malignant osteogenic tumors [10]. While osteosarcomas are overall common, only 3% of cases of osteosarcomas originate in the spine [8]. Primary osteosarcoma is most prevalent in young patients (10–20 years old), while secondary osteosarcoma (related to Paget’s disease or radiation-induced) occurs more commonly in adults [60]. Spinal osteosarcoma has a peak incidence in the fourth decade of life [21].

**Fig. 19:**
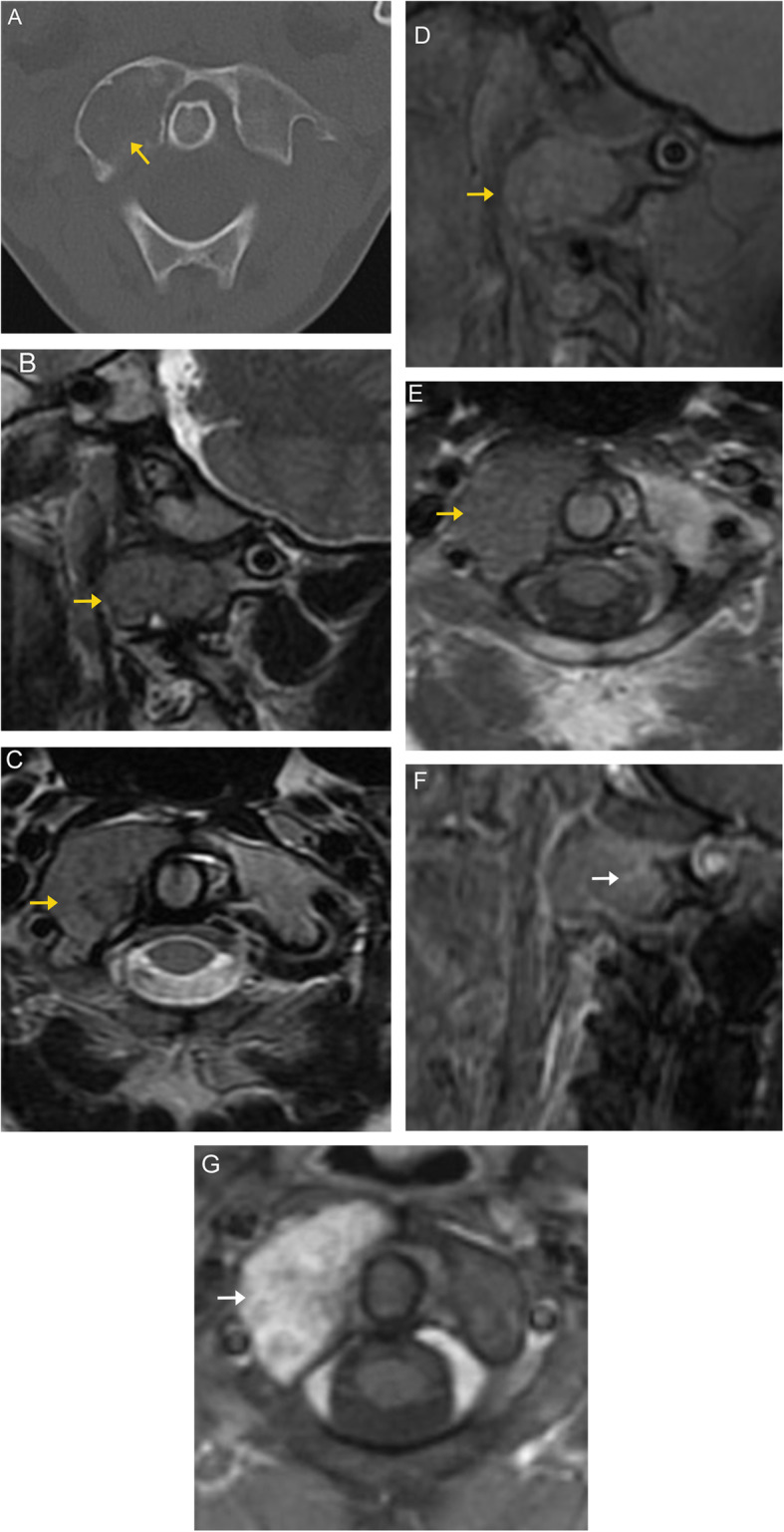
Osteosarcoma. Axial bone window CT image (**a**) demonstrates moth-eaten destruction of the right lateral mass of C1 (arrow), with a wide zone of transition along the posteromedial margin. Sagittal (**b**) and axial (**c**) T2-weighted images demonstrate the lesion to be low to intermediate in signal (arrow). The lesion is also low signal on T1-weighted images (**d**, **e**), and demonstrates avid enhancement (arrows) on post-contrast T1 sequences (**f**, **g**)

On CT imaging, these lesions appear as moth-eaten, destructive, and expansile lesions, often with a wide zone of transition [60]. On MRI, these lesions demonstrate low to intermediate signal on T1-weighted images and high signal on T2-weighted images, as well as heterogeneous enhancement. Areas of mineralization are low in signal on all sequences (Fig. 19) [8]. Spinal osteosarcomas commonly invade the spinal canal [61]. If there is no prominent soft tissue mass, osteosarcoma may be indistinguishable from an osteoblastoma. Telangiectatic osteosarcoma can have a similar appearance to an aneurysmal bone cyst. However, thick nodular cyst walls, matrix mineralization, and a more aggressive growth pattern suggest telangiectatic osteosarcoma over an aneurysmal bone cyst.

Other differential diagnoses for osteosarcoma include osteomyelitis, metastasis, and Ewing’s sarcoma.

### Ewing’s sarcoma and primitive neuroectodermal tumor

Ewing’s sarcoma and primitive neuroectodermal tumor (PNET) have a similar clinical presentation, histologic, and radiologic characteristics. Both are classified as malignant small, round cell sarcomas [10]. Immunohistochemical studies are required to distinguish these two entities. Ewing’s sarcoma may represent the primitive stage of PNET [8]. Patients typically present between ages 10 and 30, with male predominance [9]. The sacrum is most frequently involved (55% of cases) [62]. Patients typically present with pain, paresthesia, weakness, and incontinence [62]. Sacrococcygeal tumors have a worse prognosis, with only 60% local control and 25% long-term survival, probably due to larger tumor size at presentation [63].

Lytic osseous destruction is the most common radiographic finding of spinal Ewing’s sarcoma. A paraspinal soft tissue mass may also be detectable on radiographs as it is often larger than the osseous component [62].

**Fig. 20:**
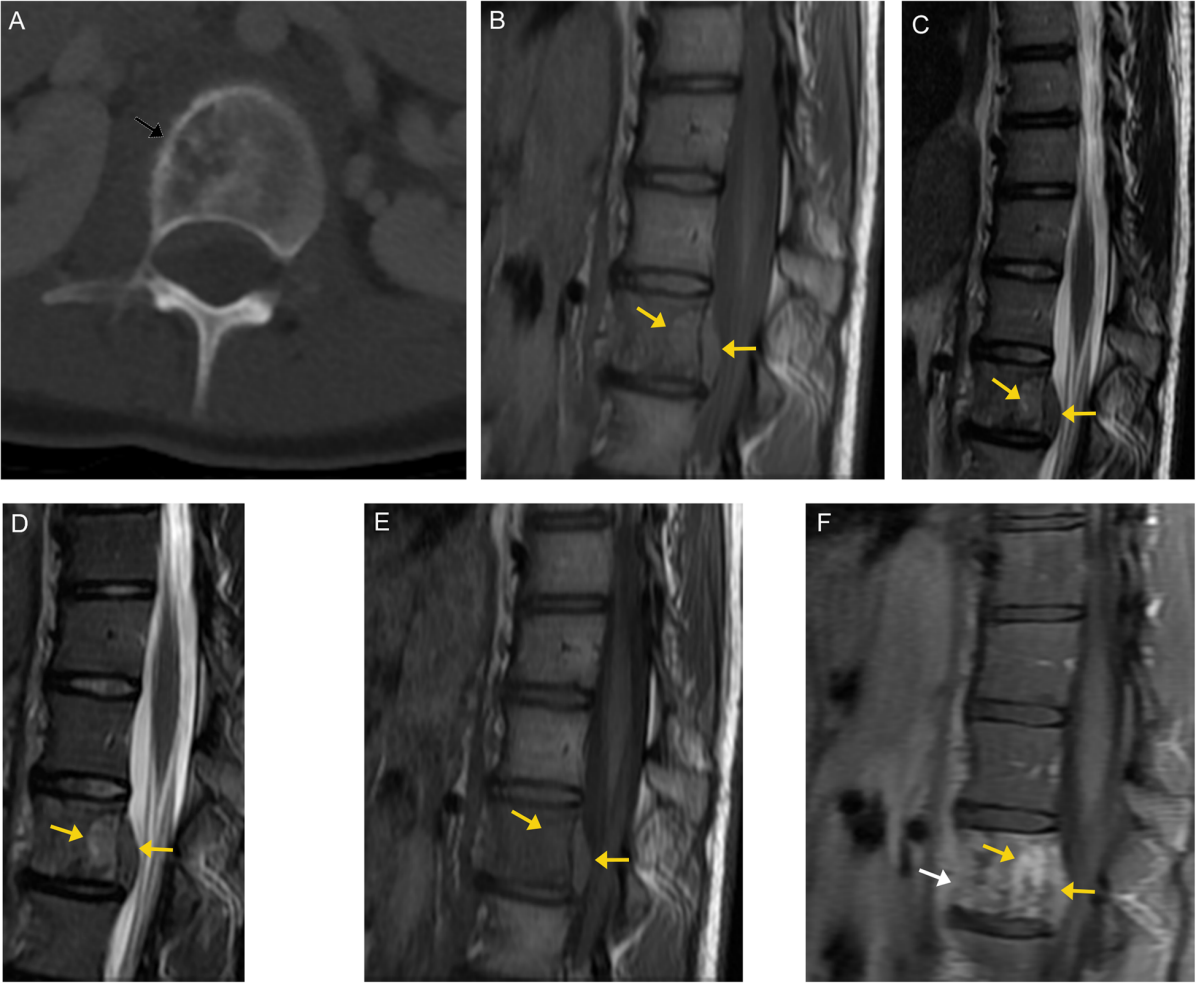
Ewing’s sarcoma. Axial CT image in the bone window (**a**) demonstrates an ill-defined lesion with destructive changes of the right vertebral body. The bone cortex has a “smudged” appearance (black arrow) due to perforation of tumor through the cortex. Sagittal proton-density image (**b**) demonstrates the ill-defined tumor as abnormal marrow signal with epidural extension (orange arrows). Sagittal T2-weighted (**c**) and T2 fat-saturated (**d**) images demonstrate variable signal within the lesion (orange arrows). This lesion demonstrates low to intermiediate T1 signal (**e**, orange arrows). On T1-weighted post-contrast imaging (**f**), there is heterogenous enhancement of the tumor (orange arrows), which is difficult to distinguish from the enhancing peritumoral edema (white arrow)

On MRI, these tumors usually have intermediate signal intensity on T1-weighted images and intermediate to high signal intensity on T2-weighted images. They may appear heterogeneous due to calcification, hemorrhage, and necrosis [8]. They are challenging to detect on T1-weighted images due to the abundant low signal red marrow in young patients, similar to the tumor itself. Post-contrast enhancement may help elucidate the tumor (Fig. 20).

Differential considerations for Ewing’s sarcoma and PNET include osteosarcoma, chondrosarcoma, and chordoma.

### Angiosarcoma

Angiosarcoma is a rare, aggressive, malignant vascular tumor according to the WHO classification [10]. It is a soft tissue sarcoma with generally poor prognosis unless diagnosed early. Angiosarcoma arises from vascular endothelial cells which show atypia and can grow along preexisting vascular channels or cavernous spaces [64]. They can also arise from poorly organized vessels. Angiosarcoma can occur throughout the body; however, the cutaneous form is most common, with the head and neck most frequently involved [65].

Osseous angiosarcoma is rare and accounts for less than 1% of primary bone tumors [64]. Vertebral angiosarcoma is extremely rare; only a few cases have been reported. The type of involvement of the spine is variable, with vertebral, paraspinal, and epidural patterns being common. The most common presenting symptom is swelling at the affected site with pain. Pathological fractures can occur.

**Fig. 21:**
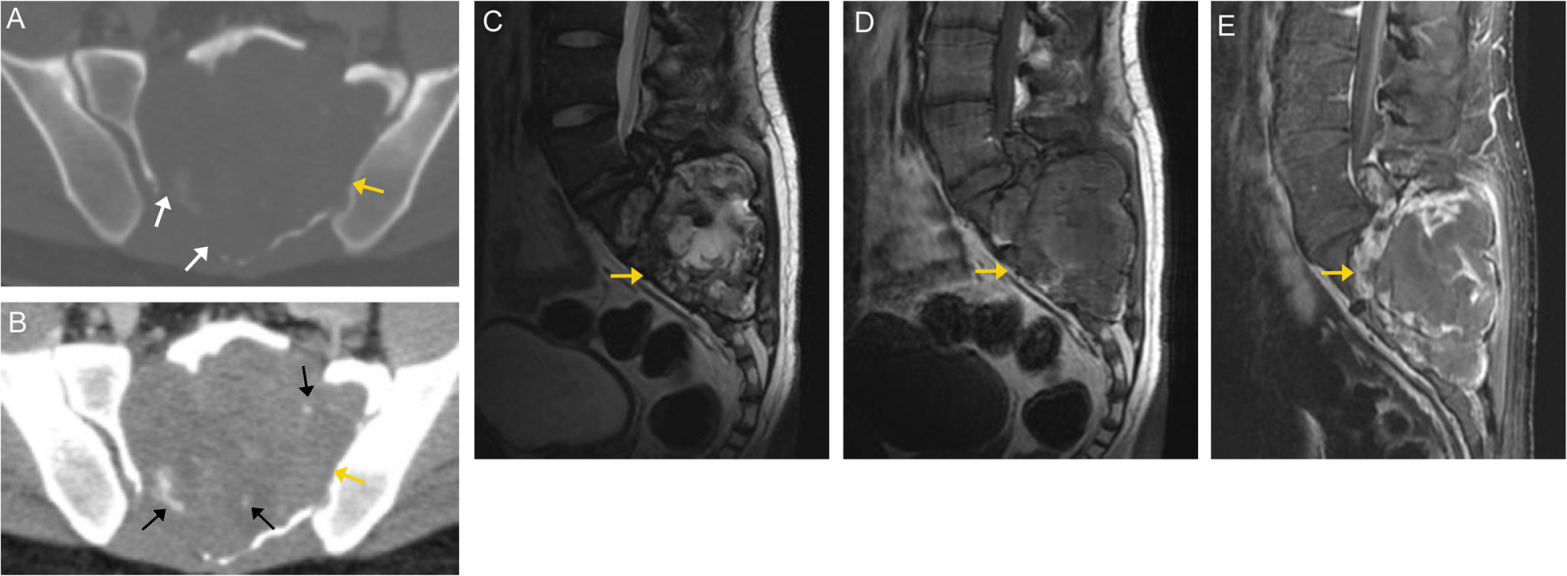
Angiosarcoma. Axial CT image in the bone window (**a**) demonstrates a large lytic lesion replacing the majority of the sacrum (orange arrow), with multiple areas of cortical erosion and destruction (white arrows). In the contrast-enhanced soft tissue window (**b**), enhancing areas are present within the mass, representing serpiginous vessels (black arrows). T2 weighted MR image (**c**) of the lumbosacral spine demonstrates a heterogeneous, destructive mass within the sacrum (orange arrows), with extension into the paraspinal soft tissues. The mass is low to intermediate signal on pre-contrast T1-weighted imaging (**d**). Post-contrast T1-weighted fat-suppressed image (**e**) demonstrates heterogeneous enhancement

Most cases on CT are lytic with cortical erosion and soft tissue extension [66]. MRI findings are nonspecific but with features of an aggressive tumor. They typically appear hypointense on T1-weighted images, heterogeneous on T2-weighted images, with heterogeneous post-contrast enhancement (Fig. 21) [64].

Given its rarity, other differential possibilities should be considered first, including aneurysmal bone cyst, osteosarcoma, and osteoblastoma. Tissue sampling is crucial for diagnosis.

## Advanced imaging techniques

Advanced imaging techniques including dual-energy CT, metal reduction MR imaging, diffusion-weighted imaging (DWI), diffusion tensor imaging (DTI), and perfusion and whole-body MR imaging are some of the newer techniques used in the evaluation of spinal osseous lesions. In the postoperative setting, dual-energy CT has proven useful in reducing streak artifacts associated with spinal fusion hardware, which can assist in the evaluation of the spinal canal and extent of possible residual tumor. Metal reduction MRI techniques have also been used with great success and can obviate the need for CT myelography while providing higher-contrast resolution and sensitivity compared to CT. DWI and DTI are imaging techniques based on microscopic diffusion of water molecules in living tissues. DWI is a powerful tool in detecting osseous tumoral involvement, particularly metastases, myeloma, and lymphoma [67, 68]. DTI examines the level of water diffusion restriction with incorporation of a directional component, which can be used to evaluate white matter tracts. This technique has not been widely used; however, it has shown potential utility in distinguishing tumors that displace the white matter tracts from those that are infiltrating [69]. An experimental study by Razek et al. using DTI has shown promise in differentiating benign from malignant vertebral compression fractures [70]. Perfusion imaging is useful in differentiating hypervascular versus hypovascular vertebral lesions. Whole-body MR imaging has a growing utility in oncologic evaluation and is a powerful tool for early diagnosis of metastases, multiple myeloma, and lymphoma [67]. Whole-body diffusion weight imaging with background body signal suppression is a novel technique that has proven to be useful in the detection of osseous and extraosseous metastases [68]. These techniques are not in mainstream use due to their limited availability and time consuming post-processing.

## Conclusion

**Fig. 22:**
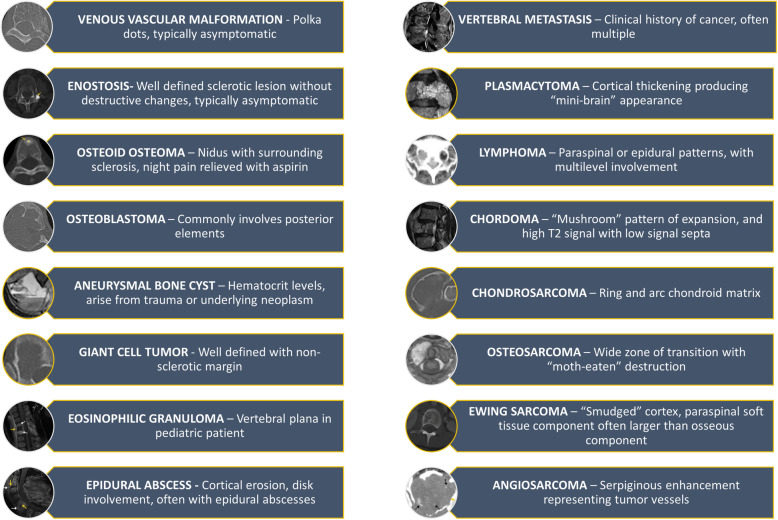
Summary of the lesions discussed in this article with their characteristic imaging and clinical findings

Spinal osseous pathology can be challenging to radiologists, specifically when presenting as solitary lesions. Metastatic disease, multiple myeloma, and lymphoproliferative diseases may be easily diagnosed on imaging given the clinical history and biochemical studies. However, other differential diagnoses must be considered when encountering a solitary spinal lesion. The lesions discussed in this article are classified as benign and malignant etiologies, followed by order of most common to least common (summarized in Fig. 22). Table 1 provides an overview of the lesions according to the WHO classification [10]. Spondylodiscitis must be considered when evaluating spinal osseous lesions, as this can be mistaken for tumor. A high index of suspicion based on history, imaging, and familiarity with these entities can help the radiologist make an accurate diagnosis, if not formulate a strong differential diagnosis and guide clinical management.

**Table 1:**
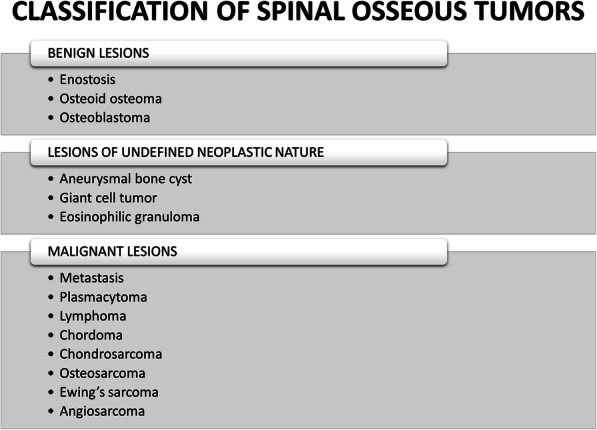
Overview of spinal osseous lesions discussed in this article based on the World Health Organization (WHO) classification

## Data Availability

Not applicable.

## Abbreviations

ABC: Aneurysmal bone cyst
CT: Computed tomography
EG: Eosinophilic granuloma
GCT: Giant cell tumor
MRI: Magnetic resonance imaging
PNET: Primitive neuroectodermal tumor
WHO: World Health Organization
